# Pneumonia in the first week after polytrauma is associated with reduced blood levels of soluble herpes virus entry mediator

**DOI:** 10.3389/fimmu.2023.1259423

**Published:** 2023-12-22

**Authors:** Noah Schaefer, Holger A. Lindner, Bianka Hahn, Roman Schefzik, Sonia Y. Velásquez, Jutta Schulte, Tanja Fuderer, Franz-Simon Centner, Jochen J. Schoettler, Bianca S. Himmelhan, Timo Sturm, Manfred Thiel, Verena Schneider-Lindner, Anna Coulibaly

**Affiliations:** Department of Anesthesiology and Surgical Intensive Care Medicine, Mannheim Institute of Innate Immunoscience (MI3), Medical Faculty Mannheim, Heidelberg University, Mannheim, Germany

**Keywords:** critical illness, herpesvirus entry mediator, immune checkpoint, immunosuppression, pneumonia, polytrauma, sepsis, systemic inflammatory response syndrome

## Abstract

**Background:**

Pneumonia develops frequently after major surgery and polytrauma and thus in the presence of systemic inflammatory response syndrome (SIRS) and organ dysfunction. Immune checkpoints balance self-tolerance and immune activation. Altered checkpoint blood levels were reported for sepsis. We analyzed associations of pneumonia incidence in the presence of SIRS during the first week of critical illness and trends in checkpoint blood levels.

**Materials and methods:**

Patients were studied from day two to six after admission to a surgical intensive care unit (ICU). Blood was sampled and physician experts retrospectively adjudicated upon the presence of SIRS and Sepsis-1/2 every eight hours. We measured the daily levels of immune checkpoints and inflammatory markers by bead arrays for polytrauma patients developing pneumonia. Immune checkpoint time series were additionally determined for clinically highly similar polytrauma controls remaining infection-free during follow-up. We performed cluster analyses. Immune checkpoint time trends in cases and controls were compared with hierarchical linear models. For patients with surgical trauma and with and without sepsis, selected immune checkpoints were determined in study baseline samples.

**Results:**

In polytrauma patients with post-injury pneumonia, eleven immune checkpoints dominated subcluster 3 that separated subclusters 1 and 2 of myeloid markers from subcluster 4 of endothelial activation, tissue inflammation, and adaptive immunity markers. Immune checkpoint blood levels were more stable in polytrauma cases than controls, where they trended towards an increase in subcluster A and a decrease in subcluster B. Herpes virus entry mediator (HVEM) levels (subcluster A) were lower in cases throughout. In unselected surgical patients, sepsis was not associated with altered HVEM levels at the study baseline.

**Conclusion:**

Pneumonia development after polytrauma until ICU-day six was associated with decreased blood levels of HVEM. HVEM signaling may reduce pneumonia risk by strengthening myeloid antimicrobial defense and dampening lymphoid-mediated tissue damage. Future investigations into the role of HVEM in pneumonia and sepsis development and as a predictive biomarker should consider the etiology of critical illness and the site of infection.

## 1 Introduction

Sepsis is associated with every fifth death globally (1), its estimated hospital mortality rate is 27% (2). Sepsis was first defined as systemic inflammatory response syndrome (SIRS) due to infection (Sepsis-1/2) (3, 4) and later as life-threatening organ dysfunction by the host response to infection (Sepsis-3) (5). In both definitions, clinical suspicion of infection marks sepsis onset. Microbiological ascertainment of infection takes hours to days and is successful in less than half of sepsis cases (6). Time-critical recognition of sepsis thus relies on clinical assessment and remains challenging (7).

Both SIRS and organ dysfunction are highly prevalent in the intensive care unit (ICU) (8), where sepsis is the leading cause of death regardless, which of the above definitions is applied, and with the lung as the primary site of infection (9, 10). SIRS and sepsis can be considered clinical phenotypes of the host response’s failure to contain local tissue inflammation after sterile and infectious tissue injury, respectively. With increasing amplitude and duration of the antagonistic pro- and anti-inflammatory responses to the injury, the capacity to restore immune homeostasis becomes limited, and the risk of secondary organ damage and infection grows (11, 12). Infection, especially pneumonia, and sepsis are frequent complications secondary to major surgery (13), polytrauma (14, 15), and burns (16).

Many characteristics of immune imbalance, particularly immunosuppression, were described for sepsis (17–19), but the roles of immune checkpoint receptors and their ligands, which normally sustain physiological self-tolerance, in tissue injury and sepsis are only starting to emerge as follows (20, 21): Increases in inhibitory immune checkpoints promote immune cell exhaustion and are thought to increase the risk of infection in critical illness. In sepsis, upregulation of programmed cell death protein 1 (PD-1) and its ligands (PD-L1 and PD-L2) on lymphoid and myeloid cells, parenchymal cells, and endothelial cells was described. Lymphocyte activation-gene-3 (LAG-3) as well as T cell immunoglobulin and mucin-domain containing-3 (TIM-3) were elevated on T cells and TIM-3 also on monocytes in sepsis but not in severe sepsis and septic shock (22). Elevated expression of cytotoxic T lymphocyte antigen-4 (CTLA-4) on T cells and of B and T lymphocyte attenuator (BTLA) and its binding partner herpesvirus entry mediator (HVEM) on lymphoid and myeloid cells in sepsis has been reported. In an unselected cohort of trauma and surgical ICU admissions sampled at, however, varying times during their stay, an over 80% BTLA positivity in the peripheral CD4+ T cell population was associated with subsequent nosocomial infection and longer hospital length of stay (LOS) (23). Post-mortem immunohistochemistry demonstrated increased HVEM-levels on airway epithelial cells but no change in lung macrophages of patients who died with active severe sepsis compared to transplant donors or lung cancer resections (24). Wakeley et al. (2020) described a relative increase in HVEM-positivity on T cells in trauma-related critical illness for patients on presentation to a level 1 trauma center, who did not develop secondary infections, compared to healthy controls. In their cross-sectional study, polytraumatized patients developing secondary infections, mainly pneumonia, were, by contrast, more similar to controls (25).

Upregulation in sepsis was also detected for co-stimulatory immune checkpoints, such as CD40 on monocytes (26–28). By contrast, cell surface expression of both co-stimulatory CD28 and inducible co-stimulatory molecule (ICOS) was reduced on a subset of CD4+ T cells positive for natural killer cell receptor 2B4+ (29).

Ectodomain shedding and alternative splicing give rise to soluble forms of immune checkpoints detectable in blood (30). While results on changes of sPD-1 and sPD-L1 levels in sepsis diverge (31–36), sepsis on ICU admission was reported to be associated with an increase in sBTLA compared to ICU controls (31, 37). sTIM-3 was reduced in ICU patients with sepsis and severe sepsis, and to a lower degree also in septic shock, compared to healthy controls (22). In a cohort of pediatric burn patients, the development of nosocomial infection was associated with elevated blood levels of sTIM-3, sCD28, and sBTLA within three days of the injury (38).

In this study, we sought to elucidate the associations of blood levels of 16 soluble immune checkpoint proteins with Sepsis-1/2 in patients with SIRS treated in a surgical ICU. For this, we determined daily immune checkpoint blood plasma concentrations together with 51 established markers of inflammation and organ dysfunction in a small group of seven polytraumatized patients with SIRS and developing pneumonia between ICU-days two and six. Immune checkpoint levels were further compared to seven clinically highly similar polytrauma controls remaining infection-free during follow-up. For two of the immune checkpoints with differences at the study baseline, baseline levels were further compared to those in unselected surgical ICU admissions with and without Sepsis-1/2 during follow-up. Results are discussed in light of the potential of immune checkpoints, particularly HVEM, for pneumonia risk estimation after polytrauma.

## 2 Methods

### 2.1 Patients and samples

The study population was a subgroup of a 99-patient cohort recruited at the anesthesiology-led interdisciplinary surgical ICU of a tertiary care hospital (University Medical Center Mannheim) between June 2017 and May 2019 as reported (39). Briefly, for cohort inclusion F.S.C. and J.J.S. evaluated consecutive patients on the first two days of their ICU admission daily at 2 PM for the presence of SIRS and Sepsis-1/2, that is SIRS driven by an infection (3, 4). Sequential Organ Failure Assessment (SOFA) scores were calculated as described (40). Patients were included at 3 PM on either day two if *SIRS* was present since ICU admission or on day one if they presented with *sepsis* but neither *severe sepsis* nor *septic shock*. The reason to not also include patients with *SIRS* already on day one was to more reliably exclude the presence of *sepsis* on inclusion in these patients and to dependably refer to them as post-injury sepsis cases if a sepsis label was subsequently assigned during follow-up. Patients were excluded in the case of chronic glucocorticoid therapy, recent organ transplant, end-stage renal failure, and neoadjuvant therapy.

EDTA-anticoagulated blood was sampled from a central venous catheter at eight-hour intervals starting at 3 PM on the day of study inclusion for a follow-up of five days or until ICU discharge or death. Samples were kept on ice, and plasma was obtained by centrifugation within 30 minutes of sampling, aliquoted, and immediately stored at -80°C.

F.S.C., J.J.S., and M.T. retrospectively adjudicated upon the presence of SIRS and all three stages of Sepsis-1/2 at all sampling time points during follow-up considering the available medical records. For 33 out of 1138 time points (2.9%), no blood sample and no definite adjudication results could be obtained. In the following, adjudication results are also referred to as expert labels and are italicized. For patients admitted due to polytrauma, T.S. retrospectively determined Injury Severity Score (ISS) values on ICU admission. All other clinical characteristics were retrieved from medical records and are reported for the time of study inclusion.

### 2.2 Comparison groups and selection of samples for analysis

**Figure 1:**
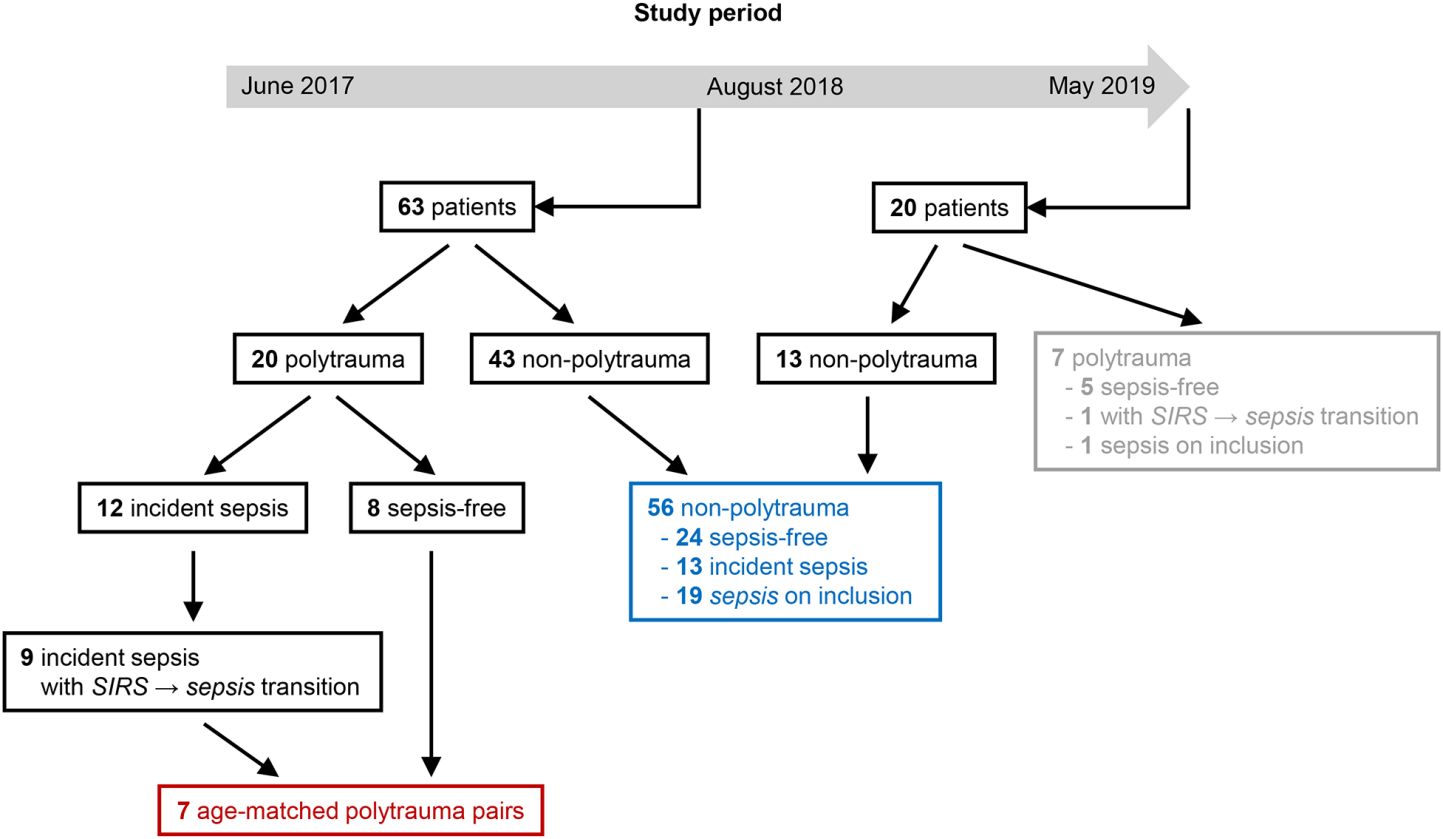
Flow diagram for selection of polytrauma (red) and non-polytrauma (surgical trauma) (blue) patients for the first and second part of the analysis, respectively, from the study population. At the beginning of August 2018, a completed 5-day follow-up series of frozen blood plasma with complete expert adjudication according to Sepsis-1/2 was available for 63 patients and an extra 20 patients until the end of the study period. Among the latter were seven polytrauma patients (gray) that were not considered.

The analyses commenced in August 2018. At that time, samples were available from 63 patients with completed follow-up and physician-adjudication (**Figure 1**). The differentiation of patients with *sepsis* from patients with non-infectious *SIRS* is particularly challenging in the critically ill, notwithstanding that *severe sepsis* and *septic shock* also require rapid diagnosis. Yet, in the first part of our study our focus was on patients, who transitioned from *SIRS* to *sepsis* on consecutive labeling time points, i.e., within eight hours, and identified nine such post-injury sepsis cases among the 63 hitherto included patients. Remarkably, these cases were all admitted due to polytrauma. Altogether 20 out of the 63 patients had SIRS due to acute polytrauma, out of which 12 developed sepsis according to Sepsis-1/2, specifically pneumonia, and nine had a direct transition from *SIRS* to *sepsis* as mentioned. Out of these and out of the eight polytrauma patients remaining infection-free during follow-up, seven case-control pairs were formed minimizing the overall mean age difference, which was 4.57 years (range 0 – 11 years).

**Figure 2:**
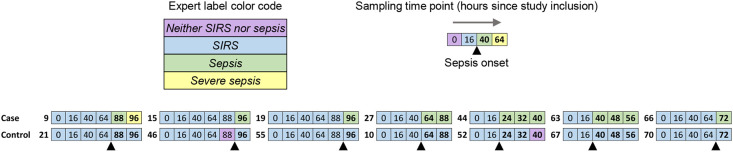
Alignment of expert label timelines for the included paired blood plasma samples. Cases and controls were polytrauma patients with a direct *SIRS*-to-*sepsis* transition, namely pneumonia development, and without a sepsis label, respectively, during follow-up. Sepsis was adjudicated by clinical experts according to Sepsis-1/2. Consecutive sampling time points are indicated, starting at 3 PM on ICU-day two (0 h), i.e. study inclusion. Sampling time points, for which samples were included in the analysis as explained in the main text, are shown. Patient identification numbers of cases and controls in a given pair are printed in bold type to the left of the corresponding aligned expert label timelines, which are color-coded as indicated. The black up-pointing triangle indicates sepsis onset. The time points for the samples obtained after sepsis onset are printed in bold type.

We first considered changes over time in the concentrations of soluble immune checkpoints and established makers of inflammation and organ dysfunction in blood plasma from the seven selected polytrauma patients, who transitioned from *SIRS* to *sepsis* during follow-up. Starting with the first sample obtained on study inclusion, i.e., 3 PM on ICU-day two, the second sample was included at 7 PM on day three and then at intervals of 24 hours until the first *sepsis* label. Subsequent samples for up to a total of five or six time points were chosen depending on plasma availability. Soluble immune checkpoints were additionally determined in plasma from the seven paired infection-free polytrauma controls on corresponding reference time points. **Figure 2** identifies the specific time points, expert labels, and patients for the 76 included samples.

In the second part of our analysis, starting at the end of the study period in May 2019, 56 unselected non-polytrauma patients with samples and expert labels available for the complete follow-up period were included (**Figure 1**). During follow-up, 24 of them remained infection-free and 13 were assigned at least one incident label for sepsis, namely, four for *sepsis*, three for *severe sepsis*, and six for *septic shock*. On study inclusion, eleven had a label for *sepsis*, six had a label for *severe sepsis* and two for *septic shock* instead of *sepsis*, i.e., for eight patients the retrospective adjudication implied a higher degree of infection severity than the original evaluation on study inclusion. The samples obtained on the inclusion of the non-polytrauma patients were assayed for selected immune checkpoints. The results are referred to as study baseline levels.

### 2.3 Magnetic bead array

Sixteen soluble immune checkpoints and 61 blood markers of inflammation and organ dysfunction were determined in cryopreserved blood plasma on a MAGPIX system (Luminex, Austin, TX, USA) with MILLIPLEX map kits (Merck Millipore, Burlington, MA, USA) according to the manufacturer’s instructions. Kits and analytes are listed in **Supplementary Table 1**. Technical duplicates were averaged and analyzed with MILLIPLEX Analyst software (Merck Millipore).

### 2.4 Protein annotation

We interrogated the GeneCards database (www.genecards.org) (41) to obtain information on the functions of plasma proteins and immune checkpoint receptor-ligand interactions, and additionally The Human Protein Atlas database (www.proteinatlas.org) (42) for cellular sources of plasma proteins.

### 2.5 Statistical analyses

#### 2.5.1 Clinical characterization

For clinical characterization, we compared continuous parameters with the t-test (Satterthwaite method), and the categorical parameters with the Chi² test or Fisher’s exact test as indicated using SAS V9.4 (Statistical Analysis System, SAS Institute, Cary, N.C., USA).

#### 2.5.2 Principle component analysis and clustered heat maps

The blood plasma concentration data of soluble immune checkpoints in the polytrauma post-injury pneumonia cases was complete. For the controls, missing values (7.7% of the data) were imputed using the SVDimpute algorithm (43) as implemented in the ClustVis tool (44) using the R/Bioconductor package *pcaMethods* (45), and values below the detection limit (0.3% of the data) were substituted by a value of one half of the lower detection limit of the corresponding immune checkpoint (46).

For blood markers of inflammation and organ dysfunction in the polytrauma post-injury pneumonia cases, concentrations above the detection limit (0.5% of the total data) were substituted by a value of 1.7 times the upper detection limit of the corresponding analyte (47). Concentrations below the detection limit (14% of the data) were substituted by a value of one-half of the lower detection limit (46). Moreover, markers with more than 40% of all concentration values below the respective detection limit (46), as well as markers with constant values in all patients, were removed from the principal component and heat map analyses, leading to a total of 51 (out of 61) analytes entering the analyses.

To get a condensed, lower-dimensional representation of the underlying data while retaining as much of the variation of the original data as possible, principal component analysis (PCA) was applied to the blood plasma protein concentrations. PCAs were conducted with centering and unit variance scaling applied to the corresponding data, where singular value decomposition was used to derive the principal components. PCA plots showing the first principal components, respectively, are provided as an output. Heat maps were created to summarize and visualize the blood plasma protein concentrations. Plasma concentrations were (row-wise) centered and unit variance was scaled across patients. For clustering, we used Euclidean distance and complete or average linkage as indicated. PCA and heat map analyses were performed via the programming language R for statistical computing (48), employing the packages PCAtools (49), scatterplot3d (50), and pheatmap (51).

#### 2.5.3 Handling of missing data in further analyses

In further analyses, soluble immune checkpoint determinations below the detection limit were treated as missing values and excluded following a conservative analytical approach.

#### 2.5.4 Measure of data dispersion

The relative dispersion of immune checkpoint plasma levels in a given patient over the follow-up period was described using the coefficient of variation (CV). CV-values in the two polytrauma subgroups were compared with the Mann-Whitney U test by applying the Bonferroni adjustment as indicated.

#### 2.5.5 Correlations

Correlations between blood plasma concentrations of soluble immune checkpoint receptors and cognate ligands were assessed with Spearman’s rank-order correlation using SigmaPlot version 11.0 (Systat Software Inc., San Jose, CA., USA) and the Bonferroni adjustment.

#### 2.5.6 Time trends

We used hierarchical linear models to evaluate potentially diverging time trends in pneumonia cases and infection-free control controls while adequately considering intra- and inter-individual variability of the repeated measures of immune checkpoint proteins in blood plasma. Analyses for each protein (dependent variable) were conducted for measurements from all time points as well as restricted to measurements taken before sepsis onset, according to Sepsis-1/2, in the cases (pre-sepsis). Whenever possible, in addition to a random intercept (starting value), a random slope and a spatial covariance structure, accounting for the sequence and irregular time intervals between measurement time points, were included in the models (see **Supplementary Table 2** for details). The latter level of complexity was unfortunately only attainable for a few pre-sepsis models. All hierarchical models further contained as independent variables a patient indicator serving as clustering variable, a time stamp reflecting the time since study inclusion for the given protein concentration, a subgroup indicator for cases and controls, and an interaction term for subgroup and time stamp for evaluation of diverging time trends. Slope and intercept from each model allowed calculation of the proportion of hourly change relative to the intercept, facilitating comparison of the relative changes across immune checkpoint proteins. The hierarchical models were fit with the mixed procedure in SAS. Bonferroni adjustment was applied to the evaluation of time trends and subgroup differences.

#### 2.5.7 Study baseline concentrations

Immune checkpoint plasma concentrations at the study baseline in the polytrauma and in different non-polytrauma patient subgroups were compared with the Kruskal-Wallis test and Dunn’s multiple comparison test using Prism 7 (GraphPad Software, San Diego, CA., USA). In addition, the Mann-Whitney U test was applied to the polytrauma cases and controls in an unadjusted analysis.

#### 2.5.8 Statistical significance

A multiple-comparison correction was applied where indicated. P-values of < 0.05 were considered statistically significant.

## 3 Results

### 3.1 Clinical characteristics of polytrauma patients

In all our seven polytrauma cases that developed sepsis according to Sepsis-1/2, infectious foci occurred in the lung. Among the microbiology test samples obtained during follow-up, bronchoalveolar lavage of four cases tested positive for Gram-negative and two additionally for Gram-positive bacteria that were also detected in the blood of one case (details on microbiology test results are given in **Supplementary Text 1**).

Demographics and clinical scores as well as hospital LOS for pneumonia cases and controls remaining free of infection during follow-up were highly similar. Patients were male except for one case. For cases and controls, the mean age ± standard deviation was 46.9 ± 15.5 and 47.7 ± 18.5 years (p = 0.927), and the mean hospital LOS was 43 ± 20 and 52 ± 43 days (p = 0.616), respectively. Hospital survival was 100% in both cases and controls. The average Charlson Comorbidity Index was 3.5 times higher in the control group (p = 0.076). There was no record of diabetes and pre-existing respiratory disease but cardiovascular disease and alcoholism in one case and two controls each (p = 1.000 for both conditions). There were also no statistically significant group differences for the ISS (p = 0.943), SOFA score (p = 1.000), the Simplified Acute Physiology Score II (p = 0.715), the Richmond Agitation-Sedation Scale (p = 0.516), and the Core-10-Therapeutic Intervention Scoring System (p = 0.101). Details on clinical scores are given in **Supplementary Table 3**.

Between ICU admission on day one and study inclusion on day two, three cases and controls each had received any type of blood component therapy (see **Supplementary Table 4** for details). Transfusions had overall been two times more frequent in controls than cases. Namely, red blood cell concentrates had been transfused in one case and three controls, fresh frozen plasma in two cases and controls each, platelet concentrates in one case and two controls, and one control had received fibrinogen replacement therapy. None of these differences was statistically significant.

On study inclusion, all polytrauma patients were mechanically ventilated, and five cases and controls each received catecholamines. **Table 1** summarizes additional selected clinical study baseline characteristics, i.e., on study inclusion. Altogether, we compared demographics, admitting department, chronic conditions, blood gas analysis, clinical chemistry, hematology, vital signs, hemodynamic and respiratory function indices, and clinical scores (see **Supplementary Table 5** for a comprehensive summary as median with interquartile range (IQR) values). Among all of these characteristics, only a higher fraction of inspired oxygen (FiO_2_) in cases (mean = 0.36 ± 0.06) than in controls (mean = 0.31 ± 0.02) reached statistical significance (p = 0.035). Medical conditions of polytrauma cases and controls on study inclusion thus appear overall highly similar.

**Table 1:** Summary of selected clinical study baseline characteristics of polytrauma post-injury pneumonia cases and age-matched infection-free polytrauma controls.

	Post-injury pneumonia (n = 7)	Infection-free (n = 7)	*p*-value
Demographics			
Age, mean (sd) (years)	47 (16)	48 (19)	0.927
Sex (male/female)	6/1	7/0	
Admitting department, n			
General surgery	2	4	0.592
Neurosurgery	1	1	1.000
Orthopedics and trauma center	4	3	1.000
Otorhinolaryngology	0	1	
Vital signs, mean (sd)			
Temperature (°C)	37.6 (0.5)	37.3 (0.5)	0.253
Respiratory rate (breaths per minute)	22.0 (2.3)	19.7 (3.0)	0.137
Heart rate (beats per minute)	96.3 (17.9)	89.7 (5.3)	0.382
Mean arterial pressure (mm Hg)	83.9 (7.4)	78.7 (12.5)	0.372
**Horovitz index, mean (sd) (mm Hg)**	256 (109)	310 (61)	0.281
Blood parameters, mean (sd)			
Lactate (mmol/L)	0.90 (0.57)	1.39 (1.00)	0.293
Glucose (mg/dL)	118 (20)	126 (19)	0.445
Sodium (mmol/L)	139 (6)	141 (4)	0.466
Total bilirubin (mg/dL)	1.32 (1.47)	1.42 (1.50)	0.897
Creatinine (mg/dL)	1.33 (1.02)	1.19 (0.48)	0.753
Hemoglobin (g/dL)	8.9 (1.5)	9.2 (1.2)	0.673
White blood cells (10^9^/L)	12.1 (2.4)	9.5 (3.6)	0.151
Platelets (10^9^/L)	164 (62)	154 (73)	0.782
CRP (mg/L)	197 (79)	163 (70)	0.411
Clinical scores, mean (sd)			
CCI	0.3 (0.5)	1.0 (0.8)	0.076
SOFA	10.3 (3.7)	10.3 (3.5)	1.000
SAPS II	28 (6)	26 (9)	0.715
RASS	-4.3 (1.5)	-4.7 (0.8)	0.516
Core-10-TISS	18 (5)	22 (3)	0.101
**Hospital mortality, n (%)**	0 (0)	0 (0)	

Sd, standard deviation. CRP, C-reactive protein. CCI, Charlson Comorbidity Index. SOFA, Sequential Organ Failure Assessment Score. SAPS II, Simplified Acute Physiology Score II. RASS, Richmond Agitation-Sedation Scale. Core-10-TISS, Core-10-Therapeutic Intervention Scoring System. Comparisons were performed with the t-test (Satterthwaite method) for continuous parameters, with the Chi² test for categorical parameters, and with Fisher’s exact test for proportions. Patients 52 and 70 in the control group had two referring departments listed, namely surgery/otorhinolaryngology and surgery/orthopedics and trauma center, respectively.

### 3.2 Soluble immune checkpoints and blood markers of inflammation and organ dysfunction in polytraumatized patients developing pneumonia

**Figure 3:**
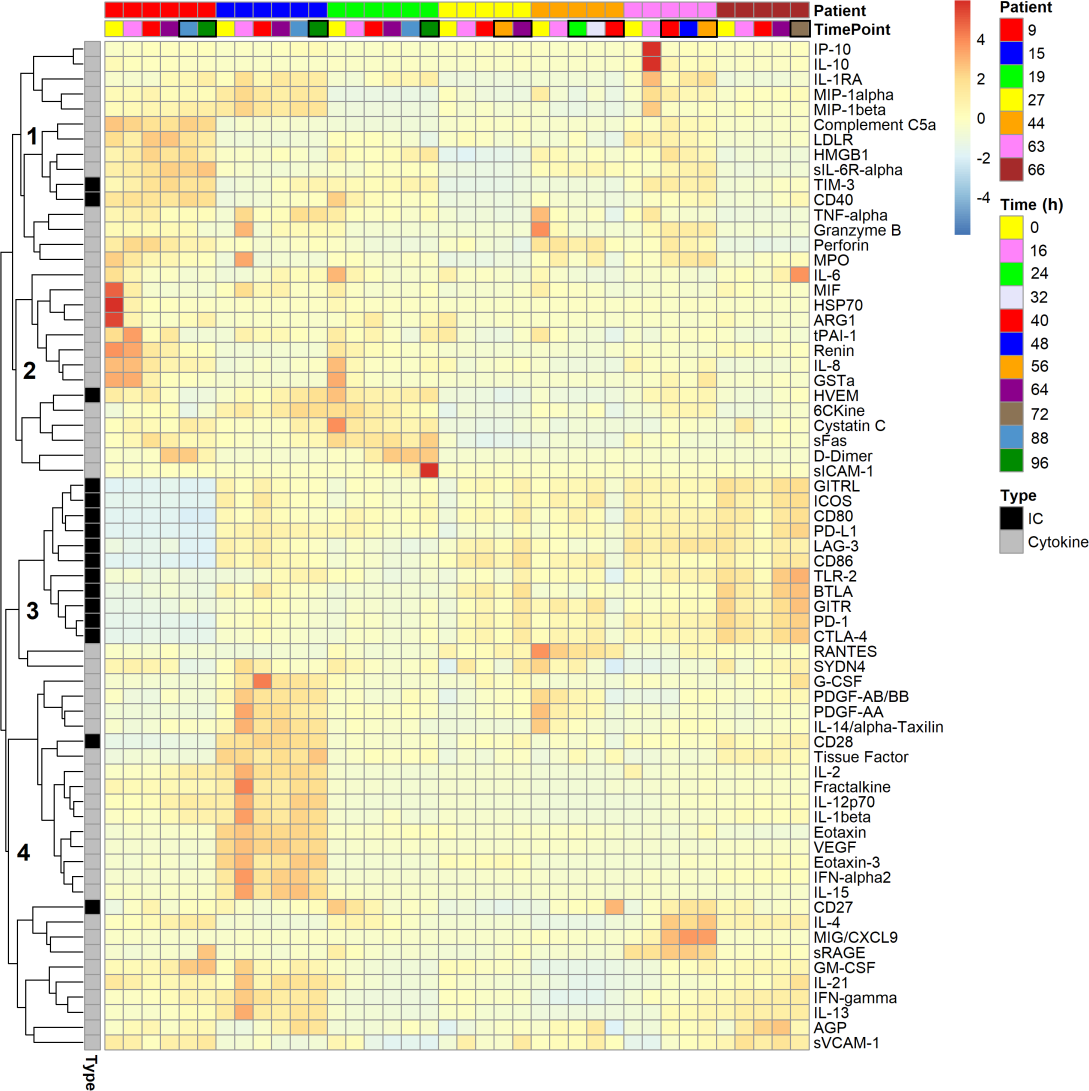
Heat map of 16 soluble immune checkpoints and 51 inflammatory and organ dysfunction marker levels in the blood plasma of seven polytraumatized patients developing pneumonia. Tiles are arranged horizontally by consecutive inclusion and by sample time point (cf. **Figure 2**). Black boxes are drawn around tiles of time points, on which patients were adjudicated septic by clinical experts according to Sepsis-1/2. The lung constituted the infectious focus in all cases. Analytes are hierarchically clustered using Euclidean distance and complete linkage. Four subclusters, 1–4, are indicated. IC: Soluble immune checkpoint proteins; Cytokine: Markers of inflammation and organ dysfunction.

For simplicity, the prefixed “s” to indicate “soluble” is omitted from the immune checkpoints’ protein symbols, and markers of inflammation and organ dysfunction are collectively referred to as cytokines. **Figure 3** summarizes the time series of blood plasma levels for 16 immune checkpoints and 51 cytokines for the seven polytrauma cases, who developed pneumonia during follow-up, as a heat map. The majority of the variance is seen between individual patients, which is consistent with a fair grouping of the samples by patient in a PCA (**Supplementary Figure 1**). Four subclusters (labeled 1–4) could be distinguished. Subclusters 1 and 2 featured two (TIM-3 and CD40) and one (HVEM) immune checkpoints, respectively, together with mainly, but not exclusively, myeloid cell-expressed/dependent cytokines and markers of polymorphonuclear neutrophil degranulation and cell death. Respective cytokines in subcluster 1 included IP-10, IL-10, IL-1 receptor antagonist (IL-1RA), MIP-1α, MIP-1β, tumor necrosis factor α (TNF-α), and in subcluster 2, IL-6, MIF and IL-8. Notably, most of them display chemotactic activities, namely, IP-10, IL-1RA, MIP-1α, MIP-1β, MIF, IL-8, and additionally complement C5a in subcluster 1. Markers of degranulation and cell death in subcluster 1 were LDLR, HMGB1, granzyme B, perforin, and myeloperoxidase (MPO), and in subcluster 2, HSP70 and ARG1. Subcluster 3 combined 11 out of the 16 immune checkpoints, mainly due to overall low and high levels in patients 9 and 66, respectively. Subcluster 4 was largely characterized by markers of endothelial activation/vascular trauma, i.e., G-CSF, PDGF, tissue factor, fractalkine/CX3CL1, VEGF, sRAGE, GM-CSF and sVCAM-1, as well as coordination of acute tissue inflammation and adaptive immune response, i.e., IL-2, IL-12p70, IL-1β, eotaxin/CCL11, eotaxin-3/CCL26, IL-15, IL-4, MIG/CXCL9, IFN-γ, IL-13 and AGP. It additionally contained the immune checkpoints CD27 and CD28.

### 3.3 Soluble immune checkpoints in polytrauma pneumonia cases and infection-free controls

#### 3.3.1 Cluster analysis and correlations

**Figure 4:**
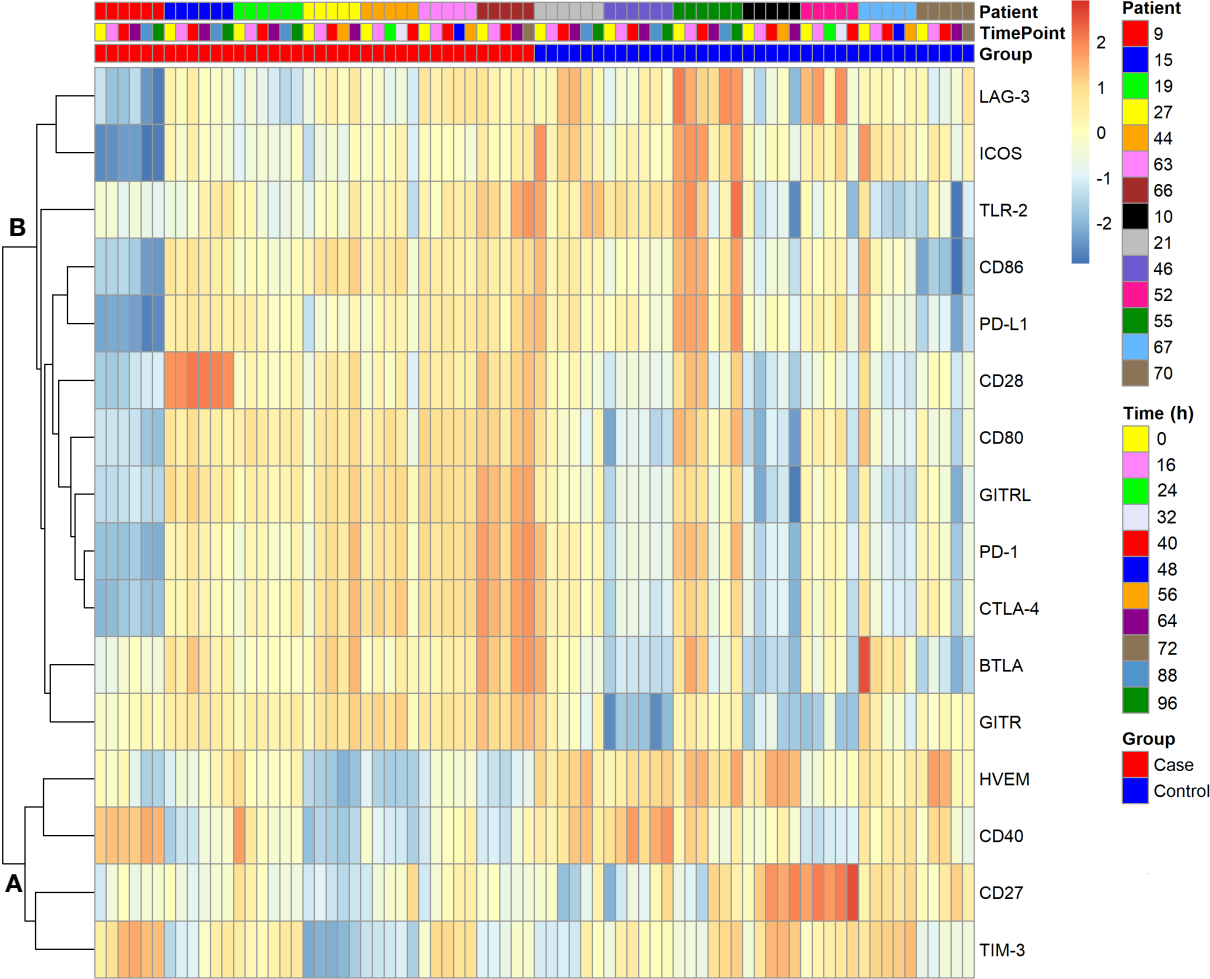
Clustered heat map of soluble immune checkpoint levels in seven polytrauma post-injury pneumonia cases and matched infection-free controls. Data were log-transformed. Analytes are hierarchically clustered using Euclidean distance and average linkage. Cases and controls each are arranged by consecutive inclusion and sampling time point (cf. **Figure 2**). Two subclusters, A and B, are indicated.

**Figure 4** shows a clustered heat map representation for the 16 immune checkpoint plasma levels in the polytrauma cases and controls. The smaller of two main checkpoint subclusters, subcluster A (TIM-3, CD27, CD40, HVEM), was characterized by rather low concentrations throughout four out of the seven cases (patients 15, 27, 44, and 66). In the larger subcluster B (GITR, BTLA, CTLA-4, PD-1, GITRL, CD80, CD28, CD86, PD-L1, TLR-2, ICOS, LAG-3), patients 15, 63 and 66 featured rather high and patient 9 low levels across all 12 immune checkpoints. In the following figures and tables, immune checkpoints are arranged in the order specified by this clustering result.

**Figure 5:**
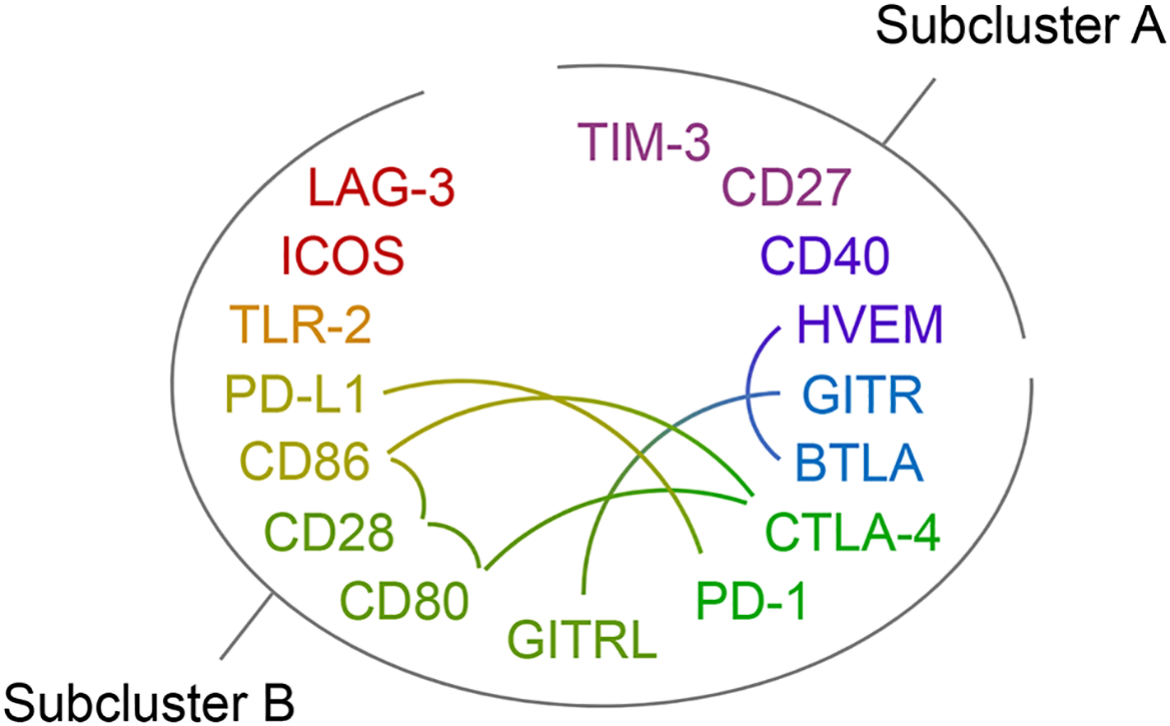
Immune checkpoint receptor-ligand pairs. The protein symbols of the 16 immune checkpoints analyzed in this study are arranged in an oval and by the clustering result displayed in **Figure 4** with subclusters A and B indicated. The rainbow color scheme reflects adjacency in the dendrogram of the cluster analysis. Reported receptor-ligand pairs among them are identified by connecting lines. Supporting literature for these receptor-ligand interactions is assembled in **Supplementary Table 6**.

Due to their functional relations, blood plasma concentrations of immune checkpoint receptors and their cognate ligands may correlate. **Figure 5** indicates reported receptor-ligand interactions among the measured immune checkpoints, six within subcluster B and one between subclusters A (HVEM) and B (BTLA) from **Figure 4** (**Supplementary Table 6** lists supporting literature for these interactions). Treating each sample as independent, the blood plasma levels of these putative binding partners showed positive correlations with overall high statistical significance in both cases and controls, except for HVEM and BTLA, which correlated in neither subgroup (**Supplementary Figure 2**). The latter agrees with their different subcluster memberships. Within subcluster A, HVEM however correlated positively with TIM-3 in cases, with CD40 in both cases and controls, and in neither subgroup with CD27 (**Supplementary Figure 3**).

#### 3.3.2 Global differences between polytrauma cases and controls

Subcluster profiles within the control group appeared overall more heterogeneous than in the cases, both within and between patients (**Figure 4**). To more closely assess the differences in the relative dispersion of immune checkpoint concentrations in the blood of cases compared to controls, we calculated the CV-values for each checkpoint and patient over the follow-up period (**Supplementary Figure 4A**). Except for CD40 and GITR, median CV-values were higher for controls than cases. This difference reached statistical significance in an adjusted analysis for CD80 with a 1.81-fold higher median CV-value in controls than cases. The median across all 16 immune checkpoint median CV-values for controls was 24.6% compared to 16.4% for cases (p = 0.001) indicating overall more stable immune checkpoint levels in cases (**Supplementary Figure 4B**). PCA based on the immune checkpoint plasma concentrations further suggests more similarity within than between the two patient subgroups (**Supplementary Figure 5**). Overall, samples from the same patient tended to group together.

#### 3.3.3 Differences in time trends between polytrauma cases and controls

**Figure 6:**
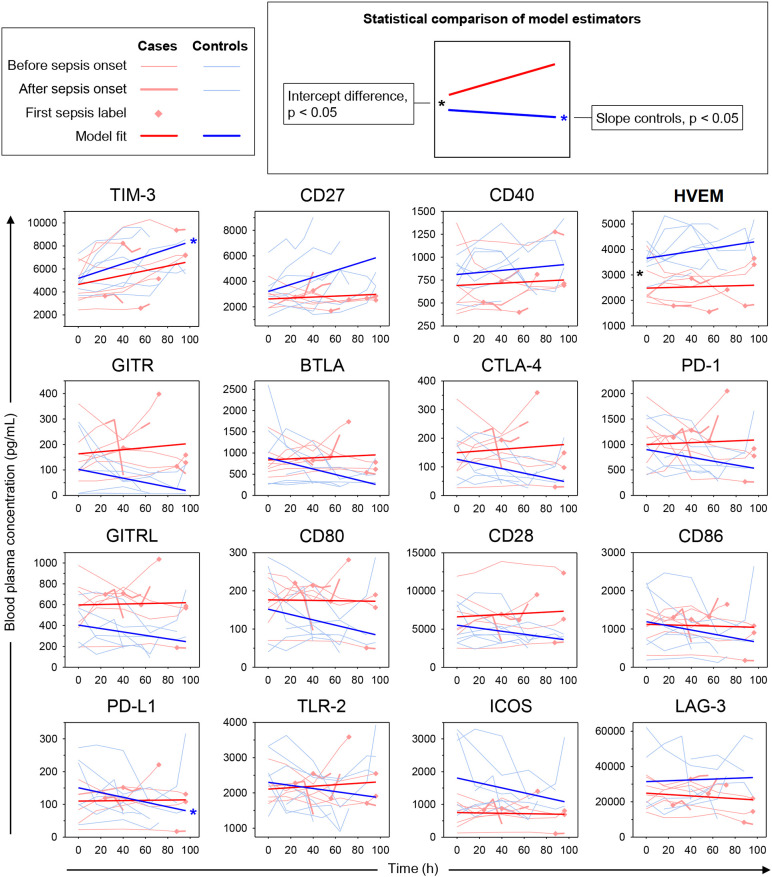
Time trends in soluble immune checkpoints in polytrauma post-injury pneumonia cases and matched infection-free controls. Sepsis was adjudicated according to Sepsis-1/2 by clinical experts. The lung constituted the infectious focus in all cases. Trajectories of measured blood plasma concentrations are overlaid with estimated time trends from hierarchical linear models (see legend on the top-left). *p < 0.05 after Bonferroni adjustment.

We next built hierarchical linear models of time trends in soluble immune checkpoint levels in the blood plasma of cases and controls and compared model estimates for slope (change over time) and intercept (study baseline value). **Table 2** summarizes model results, and **Figure 6** displays measured values with model estimates. Overall, we estimated smaller changes over time in cases than controls except, potentially, for LAG-3. Estimated slopes, however, only reached statistical significance in controls with positive and negative signs, respectively, for TIM-3 (p = 0.030) and PD-L1 (p = 0.029). There was, however, no statistically significant subgroup difference in estimated slopes. For the estimated intercept, a 1.47-fold higher value for HVEM in controls than cases (Δ, 1174 pg/mL [95% CI, 443 – 1904 pg/mL], p = 0.036) was the only statistically significant subgroup difference.

With the potential of soluble immune checkpoints to support clinical recognition of pneumonia in mind, we also restricted the model to the measurements before pneumonia onset (**Supplementary Table 7**). Again, we estimated overall smaller slopes for cases than controls except for LAG-3. A positive estimated slope for CD27 in controls of 26.76 pg/mL/h reached statistical significance (p = 0.020) and also significantly differed from the negative slope in cases of -6.96 pg/mL/h (p=0.035). Similar to the complete follow-up (**Table 2**), the estimated intercept for HVEM was 1.51-fold higher in controls than in cases (Δ, 1256 pg/mL [95% CI, 499 – 2012 pg/mL], p = 0.030). Additionally, the estimated intercept for ICOS was 2.49-fold higher in controls than cases (Δ, 1092 pg/mL [95% CI, 400 – 1783 pg/mL], p = 0.044).

**Table 2:** Estimates for intercept (study baseline value) and slope (hourly change) from hierarchical linear models of time trends of soluble immune checkpoints in blood plasma of polytrauma patients throughout follow-up and statistical evaluations after Bonferroni adjustment.

Immune Check-point	Polytrauma post-injury pneumonia cases				Polytrauma infection-free controls				Subgroup differences	
	Intercept (pg/mL)	Slope [pg/mL/h] (95% CI)	Relative hourly change [% of intercept]	P-value slope	Intercept (pg/mL)	Slope [pg/mL/h] (95% CI)	Relative hourly change [% of intercept]	P-value slope	P-value intercept difference	P-value slope difference
TIM-3	4649	20.01 (2.52 – 37.50)	0.43	0.453	5178	31.8 (14.31 – 49.29)	0.61	**0.030**	1.000	1.000
CD27	2617	3.71 (-13.70 - 21.11)	0.14	1.000	3203	27.49 (10.09 – 44.89)	0.86	0.078	1.000	**0.648**
CD40	691	0.63 (-2.45 – 3.70)	0.09	1.000	811	1.13 (-1.95 – 4.20)	0.14	1.000	1.000	1.000
HVEM	2480	1.21 (-8.83 – 11.25)	0.05	1.000	3654	6.65 (-3.39 – 16.69)	0.18	1.000	**0.036**	1.000
GITR	163	0.41 (-0.59 – 1.42)	0.25	1.000	102	-0.87 (-1.90 – 0.16)	-0.85	1.000	1.000	0.960
BTLA	838	1.21 (-4.58 – 7.00)	0.14	1.000	884	-6.51 (-12.40 – -0.62)	-0.74	0.529	1.000	0.770
CTLA-4	150	0.3 (-0.32 – 0.92)	0.20	1.000	127	-0.81 (-1.49 – -0.13)	-0.64	0.385	1.000	0.205
PD-1	1001	0.90 (-2.15 – 3.95)	0.09	1.000	900	-3.80 (-7.19 – -0.41)	-0.42	0.495	1.000	0.483
GITRL	598	0.22 (-1.29 – 1.74)	0.04	1.000	404	-1.65 (-3.33 – 0.02)	-0.41	0.839	1.000	1.000
CD80	177	-0.04 (-0.61 – 0.53)	-0.02	1.000	152	-0.70 (-1.31 – -0.08)	-0.46	0.477	1.000	1.000
CD28	6605	7.68 (-8.49 – 23.85)	0.12	1.000	5513	-19.55 (-35.99 – -3.10)	-0.35	0.379	1.000	0.218
CD86	1121	-0.78 (-4.45 – 2.88)	-0.07	1.000	1189	-5.41 (-9.34 – -1.49)	-0.46	0.124	1.000	1.000
PD-L1	110	0.04 (-0.38 – 0.45)	0.03	1.000	151	-0.73 (-1.17 – -0.28)	-0.48	**0.029**	1.000	0.234
TLR-2	2107	2.06 (-2.64 – 6.77)	0.1	1.000	2301	-4.39 (-9.25 – 0.47)	-0.19	1.000	1.000	0.980
ICOS	750	-0.55 (-6.22 – 5.13)	-0.07	1.000	1805	-7.51 (-13.49 – -1.53)	-0.42	0.290	0.201	1.000
LAG-3	24881	-38.69 (-108.20 – 30.81)	-0.16	1.000	31431	24.18 (-59.61 – 107.96)	0.08	1.000	1.000	1.000

CI, confidence interval.

The associations between elevated blood HVEM and ICOS already at the study baseline and the absence of pneumonia during follow-up, according to our time trend analyses, may have predictive value. An unadjusted statistical analysis of the baseline data indicates elevated levels in polytrauma cases compared to controls, however, only for HVEM (1.61-fold, p = 0.025). In the following, we sought to determine whether the baseline levels of HVEM, and additionally ICOS, differed in our unselected non-polytrauma patients with and without sepsis according to Sepsis-1/2.

### 3.4 Clinical characteristics of unselected non-polytrauma patients with and without sepsis according to Sepsis-1/2

**Table 3** lists clinical study baseline characteristics for the unselected non-polytrauma patients (**Figure 1**), that is surgical admissions to our ICU (see **Supplementary Table 8** for a summary of all characteristics considered as medians with IQR values). In contrast to the polytrauma patients, they showed several subgroup differences detailed in **Supplementary Text 2**. Briefly, the non-polytrauma patients were overall older than the polytrauma patients and contained more men than women in the incident sepsis compared to the sepsis-free subgroup. Mortality was highest in the incident sepsis subgroup. Baseline disease severity was highest in this subgroup as judged by SOFA, catecholamine therapy, and systolic blood and mean arterial pressure. In agreement with the presence of infection, C-reactive protein (CRP) and proportions of neutrophils were elevated and lymphocytes were reduced in the sepsis on-inclusion subgroup. There were no subgroup differences in pre-existing chronic conditions and mean hospital LOS. Microbiology test results are given in **Supplementary Text 1**.

**Table 3:** Summary of selected clinical study baseline characteristics of non-polytrauma patients and hospital mortality.

	Sepsis^a^ on inclusion (n = 19)	Incident sepsis^a^ (n = 13)	Sepsis-free^a^ (n = 24)
Demographics			
Age, mean (sd) (years)	61 (19)	66 (16)	62 (17)
Sex (male/female)	10/9	11/2	9/15*
Admitting department, n (%)			
General surgery	8 (42)	6 (46)	9 (38)
Neurosurgery	0 (0)	4 (31)^#^	10 (42)^#^
Orthopedics and trauma center	3 (16)	1 (8)	2 (8)
Otorhinolaryngology	4 (21)	1 (8)	3 (13)
Other	5 (26)	1 (8)	3 (13)
Chronic conditions, n (%)			
Diabetes	5 (26)	1 (8)	8 (33)
Respiratory disease	2 (11)	1 (8)	0 (0)
Cardiovascular disease	9 (47)	4 (31)	15 (63)
Alcoholism	1 (5)	0 (0)	1 (4)
Treatments			
Mechanical ventilation n (%)	15 (79)	12 (92)	20 (83)
Catecholamines n (%)	11 (58)*	12 (92)	13 (54)*
Vital signs, mean (sd)			
Temperature (°C)	37.1 (0.7)	37.2 (0.5)	37.1 (0.7)
Respiratory rate (breaths per minute)	19.2 (5.7)	20.7 (6.9)	18.0 (5.3)
Heart rate (beats per minute)	84.8 (16.3)	89 (22)	85.4 (17.1)
Mean arterial pressure (mm Hg)	84.5 (15.5)	76.5 (11.3)	84.6 (12.3)
**Horovitz index, mean (sd) (mm Hg)**	252 (95)	274 (86)	302 (85)
Blood parameters, mean (sd)			
Lactate (mmol/L)	1.12 (0.54)	1.65 (2.48)	1.32 (0.73)
Glucose (mg/dL)	132 (39)	143 (38)	154 (54)
Sodium (mmol/L)	142 (8)	141 (4)	141 (5)
Total bilirubin (mg/dL)	0.68 (0.47)	0.57 (0.26)	0.77 (0.69)
Creatinine (mg/dL)	1.66 (1.45)	1.34 (0.96)	1.07 (0.58)
Hemoglobin (g/dL)	9.8 (1.6)	8.9 (1.0)	9.0 (1.1)^#^
White blood cells (10^9^/L)	13.3 (5. 8)	10.7 (7.1)	12.8 (4.3)
Platelets (10^9^/L)	231 (92)	176 (88)	155 (84)
CRP (mg/L)	225 (116)	151 (86)	116 (78)^#^
Clinical scores, mean (sd)			
CCI	2.3 (1.8)	2.2 (2.2)	2.2 (2.1)
SOFA	6.4 (2.3)*	9.8 (3.9)	7.1 (3.3)
SAPS II	36 (10)	38 (12)	34 (9)
RASS	-1.8 (2.0)	-2.3 (2.7)	-1.7 (1.7)
Core-10-TISS	16 (7)	18 (6)	14 (6)
**Hospital mortality, n (%)**	4 (21)*	8 (62)	4 (17)*

a Sepsis-1/2.

Sd, standard deviation.

CCI, Charlson Comorbidity Index.

SOFA, Sequential Organ Failure Assessment Score.

SAPS II, Simplified Acute Physiology Score II.

RASS, Richmond Agitation-Sedation Scale.

Core-10-TISS, Core-10-Therapeutic Intervention Scoring System.

Catecholamines include adrenalin, noradrenalin, dobutamine, and combined treatment.

Total bilirubin values were missing three-times in the sepsis on admission and once each in the incident sepsis and sepsis-free subgroups.

Values for creatinine, white blood cells, platelets and C-reactive protein (CRP) were missing twice each in the sepsis on-inclusion subgroup.

Further selected clinical characteristics mentioned in the text are included in **Supplementary Table 7**.

Comparisons were performed with the t-test (Satterthwaite method) for continuous parameters, with the Chi² test for categorical parameters, and with Fisher’s exact test for proportions.

* (p < 0.05 for the comparison against incident sepsis).

^#^ (p < 0.05 for the comparison against sepsis on inclusion).

### 3.5 Study baseline levels of HVEM and ICOS in polytrauma and non-polytrauma ICU admissions

**Figure 7:**
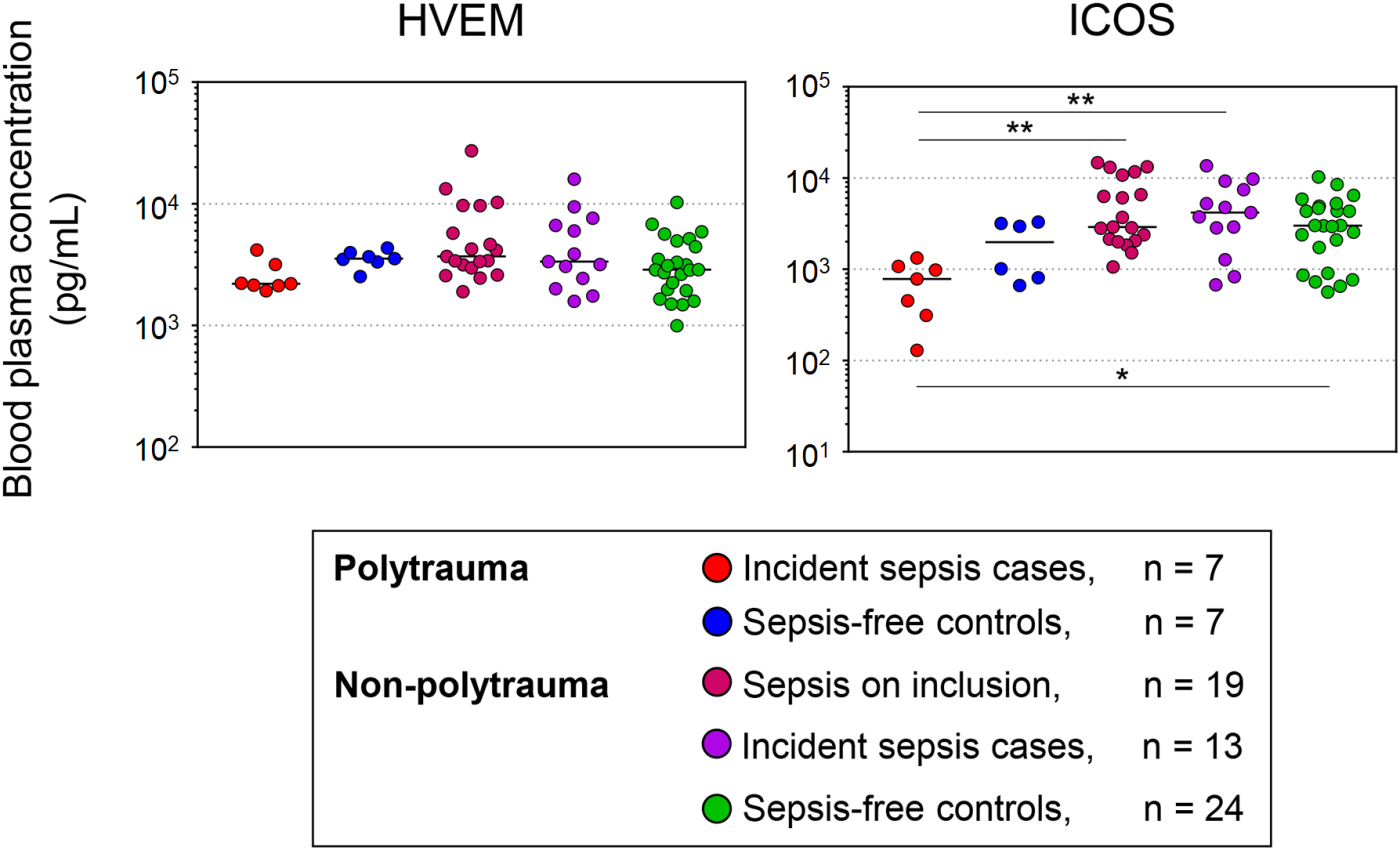
Study baseline blood levels of HVEM and ICOS in polytrauma and non-polytrauma patients with and without sepsis according to Sepsis-1/2 based clinical expert adjudication. Concentrations are plotted on a log scale. Differences across all groups were assessed with the Kruskal-Wallis test followed by Dunn’s test for multiple comparisons. * p < 0.05, ** p < 0.005.

**Figure 7** summarizes the comparisons of the study baseline levels of HVEM and ICOS across all polytrauma and non-polytrauma subgroups with correction for multiple testing. The absence of any difference among the three larger non-polytrauma subgroups is notable. The main differences consisted of overall lower levels of ICOS in the polytrauma cases than in all non-polytrauma subgroups.

## 4 Discussion

### 4.1 Study design and measurements

The recognition that immunosuppression is a major risk factor for infection and sepsis in the critically ill has put a spotlight on immune checkpoints as potential predictive and diagnostic sepsis markers in this patient population (52). Many immune checkpoints are detectable in soluble form in peripheral blood. We asked whether their concentrations in patients admitted to our interdisciplinary surgical ICU with trauma-induced SIRS were associated with the development of infection-triggered SIRS, i.e., *sepsis* according to Sepsis-1/2, during the first week. The emulation of this clinical challenge, i.e., the early differential diagnosis of sepsis in this setting, with high fidelity in an observational study is complicated by the strong heterogeneity of the patient population and the presentation of the syndrome. Moreover, we previously determined that 93% of the patient days in our ICU were associated with a SOFA > 2 compared to 68% with SIRS, regardless of infection (8).

The first part of the current study was focused on the particularly challenging differential diagnosis of an infection as a trigger of systemic inflammation in patients with pre-existing SIRS, which we found to be common in polytrauma patients developing pneumonia during the first week in the ICU (**Figure 1**). We included polytrauma admissions, who experienced a direct transition from *SIRS* to *sepsis*. For seven such incident cases, we identified seven polytrauma patients remaining infection-free during follow-up that were highly similar with regard to clinical characteristics. These included the severity of the traumatic injury, degree of organ dysfunction, and other risk factors, as well as treatment intensity (**Supplementary Table 5**). Starting on ICU-day two, one blood plasma sample was included daily for analysis until the onset of *sepsis* (pneumonia) and additional samples thereafter (**Figure 2**). Therein, 16 immune checkpoints were quantified by multiplex bead array. For each of them, the absolute concentrations determined were on essentially the same levels as reported previously by Wu et al. (2021) for polytraumatized patients on day three in the ICU using the same commercial assay (53), which supports the consistency of this analytical method.

### 4.2 Immune checkpoints after polytrauma

We first considered the blood plasma immune checkpoint profiles in the context of the profiles of 51 established blood markers of inflammation and organ dysfunction detectable in the polytrauma post-injury pneumonia cases (**Figure 3**). TIM-3 as well as CD40 in subcluster 1, both reportedly upregulated on sepsis monocytes (22, 26–28), and HVEM in subcluster 2, previously shown to mediate enhancement of *in vitro* bactericidal activities of monocytes and neutrophils (54), indeed clustered with markers of myeloid cell activation including cytokines, mainly chemokines, and markers of degranulation and cell death. Notably, eleven checkpoints essentially defined the distinct subcluster 3. The remaining two checkpoints, the T cell-expressed co-stimulatory molecules CD28 and CD27, were contained in subcluster 4 together with proteins mainly originating from activated endothelium/vascular trauma and inflamed tissue and active in the transition to an adaptive immune response. This clustering result suggests that immune checkpoint profiles in blood after polytrauma are related to distinct submechanisms of the inflammatory process.

Next, we subjected the blood levels of the 16 immune checkpoints in both the polytrauma cases and time-matched samples from infection-free controls (**Figure 2**) to a cluster analysis obtaining two well-separated subclusters, A and B (**Figure 4**, **Figure 5**). Here, CD27 from subcluster 4 of the above case-restricted analysis (**Figure 3**) merged with the smaller subcluster A, containing TIM-3 and CD40 from subcluster 1 as well as HVEM from subcluster 2, and CD28 with the main subcluster B, otherwise comprised of the eleven subcluster 3 immune checkpoints. A comparison of the relative variance of immune checkpoint levels confirmed the impression from the heat map in **Figure 4** that levels were overall more stable in cases than controls (**Supplementary Figure 4**). Apparently, more pronounced and dissimilar dynamics for CD27 and CD28 in the controls compared to cases accounted for their divergent subcluster memberships in **Figure 4** against the first case-restricted cluster analysis in **Figure 3**.

PCA, based on the immune checkpoint plasma concentrations, confirmed higher overall similarities of samples within than between the 14 polytrauma patients (**Supplementary Figure 5**). But it also suggested a fair separation of cases and controls. To gain further insight into pneumonia-associated differences in the time trends of immune checkpoint levels in cases and controls we used hierarchical linear models accounting for intra- and inter-individual variability in repeated measurements. The results corroborated a difference only for CD27 and consisted in a steeper estimated increase before the time of pneumonia onset in controls than in cases (**Supplementary Table 7**). Throughout follow-up, relatively high positive slopes in the controls, reaching statistical significance for TIM-3, was indeed a defining feature of subcluster A compared to negative control slopes in subcluster B (except for LAG-3) reaching statistical significance for PD-L1 (**Figure 6**, **Table 2**). In both subclusters, immune checkpoint levels in cases overall trended towards lesser changes over time than in controls. The significant rise in TIM-3 in polytrauma cases, despite no significant difference from the controls, may correspond to elevated levels of this protein in pediatric burn patients developing nosocomial infection (38).

Another common feature of subcluster A-immune checkpoints, in addition to positive control slopes, consisted of higher levels in controls than in cases throughout follow-up. This was most pronounced for HVEM and was reflected by a significantly higher estimated study baseline value (intercept) both when considering all measurements (**Table 2**) and the measurements before pneumonia onset only (**Supplementary Table 7**). In the latter subanalysis, this was also the case for ICOS, where controls however trended towards a negative slope, potentially mitigating the difference between case and control levels over time. HVEM, by contrast, if any slightly increased over time in controls and appeared relatively stable in cases (see also **Supplementary Figure 4**).

### 4.3 HVEM signaling

Among all the above-described characteristics of immune checkpoint blood levels in polytrauma patients, the consistently reverse relation between HVEM levels and pneumonia incidence makes HVEM a candidate predictive marker for pneumonia in these patients. Higher measured study baseline levels for HVEM in controls than cases reached statistical significance in an unadjusted analysis. This and overall similar case and control levels of HVEM’s main binding partner BTLA in our study, however, contrast reportedly elevated levels in sepsis for HVEM on monocytes and granulocytes and for BTLA on CD4-positive T cells, monocytes and granulocytes (23, 55), as well as higher post-mortem HVEM levels in the respiratory epithelium and unchanged levels on lung macrophages (24). Contrarily, the similarly reverse relation between sepsis incidence and T cell positivity for HVEM after polytrauma reported by Wakeley et al. (2020) (25) matches our finding for HVEM blood levels. In their report, five out of seven post-injury sepsis cases developed pneumonia compared to all seven in our study. This may point towards the lung as a source of elevated HVEM in the controls and a role of HVEM in lowering the risk of secondary infection in this tissue as further discussed below.

The biology of HVEM as both a ligand and a receptor (56) and its role in sepsis (57) were reviewed previously. Briefly, HVEM is widely expressed in adult tissues with the highest levels in the spleen, blood lymphocytes, and myeloid cells. Its membrane-bound binding partners BTLA, CD160, and LIGHT (lymphotoxin-like, inducible expression, competes with herpes simplex virus glycoprotein D for HVEM, a receptor expressed by T lymphocytes), as well as soluble LTα (lymphotoxin α), are expressed by lymphoid cells and LIGHT additionally by myeloid cells. The binding of HVEM to LTα and LIGHT leads to NFκB activation and immune stimulation in the HVEM-expressing cell. As a ligand, HVEM however induces immunosuppression in BTLA- and CD160-expressing cells. In the same naïve T cell, inhibitory signaling through BTLA overrules concomitant stimulatory signaling through HVEM upon receptor-ligand engagement.

Given its complexity, the net outcome of HVEM signaling is highly context-dependent. Its role in fine-balancing immune tolerance and activation, unsurprisingly, makes HVEM a two-edged sword in sepsis. In various mouse models of intestinal and respiratory bacterial infections, protective mucosal barrier function depended either on the stimulatory HVEM-LIGHT axis or the inhibitory HVEM-CD160 axis but did not involve BTLA in either case (57). Notably, siRNA-mediated knockdown of upregulated pulmonary HVEM in a double-hit mouse model of acute lung injury (ALI) by hemorrhagic shock followed by polymicrobial abdominal sepsis dampened myeloid cell-mediated lung damage and improved survival (58). The latter points towards a relatively high importance of the immune stimulatory activity of HVEM in the lung.

We cannot directly extrapolate from our patient subgroup differences in blood levels of immune checkpoints to their activation status in blood cells or tissues. Yet, controlled ectodomain shedding is fundamental to the regulation of cell surface receptors and ligands (59). It is reasonable to assume that it contributes to these differences and adds to the high positive correlations of the blood levels for immune checkpoint receptor-ligand pairs within subcluster B in cases and controls (**Supplementary Figure 2**). Levels of BTLA from subcluster B, however, showed no correlation with its ligand HVEM from subcluster A. As mentioned above, both elevated HVEM levels in T cells (25) and, even more so, in blood (this study) were associated with the development of secondary pneumonia after polytrauma. The essential role of LIGHT but not BTLA in the reported HVEM-dependent protection of respiratory mucosal barrier function in an animal model of Chlamydia psittaci respiratory tract infection (60) would be consistent with an involvement of the immune-stimulatory HVEM-LIGHT axis rather than inhibitory HVEM-BTLA axis. Immune-mediated lung damage after polytrauma and hemorrhage, in particular, is driven by thrombo-inflammation and involves the complement cascade and infiltration of the lung with activated myeloid cells (61, 62). The clustering of elevated blood HVEM with blood markers of these inflammatory processes, such as complement C5a and MPO (subclusters 1 and 2 in **Figure 3**), thus indicates that HVEM may escape from the inflamed lung into the blood after polytrauma (this study). This may particularly be exemplified by patient 9 showing the overall highest plasma levels of subcluster 1 and 2 proteins (**Figure 3**).

Taken together, HVEM contributes to myeloid cell-mediated lung damage in the double-hit ALI model (58) and to respiratory mucosal barrier integrity, through the HVEM-LIGHT axis, in the Chlamydia psittaci infection model (60). Both reports are consistent with an interaction between HVEM, upregulated in the respiratory epithelium (24) and on T cells (25), and LIGHT on infiltrating myeloid cells. This both enhances antimicrobial defense but also drives lung inflammation. Wakeley et al. (2020), nevertheless, explained higher HVEM levels on T cells in their polytrauma controls than in post-injury pneumonia cases with a role of HVEM signaling in lymphocytes in maintaining an appropriate immune response after critical tissue trauma (25). This interpretation may also, at least partially, apply to the corresponding HVEM blood profile in our polytrauma patients.

We feel however that, in addition to these pro-inflammatory properties of HVEM, we also need to consider its immunosuppressive functions that may balance its lung damage potential. Interestingly, Mintz et al. (2019) reported that, in a mouse model, B-cell-expressed HVEM at the immunological synapse restricts T cell help during the germinal center (GC) response through engaging BTLA on T cells (63). This limits T cell receptor signaling and assembly of preformed intracellular CD40L (CD40 ligand) into the synapse. Reduced recruitment of CD40 on B cells, in turn, then destabilizes the synapse. Therefore, it is tempting to speculate that T follicular helper (Tfh)-like cells, that infiltrate the inflamed lung and express Tfh-markers CD40L and IL-21 (64), interact with naïve B cells in bronchus-associated lymphatic tissue through this particular HVEM-BTLA axis after polytrauma. Despite unchanged blood levels of BTLA, this interaction may still lead to the release of HVEM and CD40 from the B cells as a counter-regulatory and lung protective mechanism and accounting for the elevated HVEM and CD40 levels in blood. These two, indeed, correlate positively in cases and controls (**Supplementary Figure 3**). On that note, the increasing levels of CD27 in our polytrauma controls compared to cases until the time of pneumonia onset (**Supplementary Table 7**) likely reflect continued activation of T cells (65) and thus potentially, the need for their effective control to limit immune-mediated lung damage. Uncontrolled T cell and endothelial activation may dominate, for instance, in patient 15 featuring the overall highest levels of the corresponding markers in subcluster 4 (**Figure 3**).

### 4.4 Immune checkpoints after surgical trauma

We further assessed whether the development of sepsis according to Sepsis-1/2 within the first week in the ICU was associated with low HVEM and potentially ICOS in blood at the study baseline only in polytrauma patients or also in our unselected surgical admissions (non-polytrauma patients) (**Figure 1**). Besides subgroups of non-polytrauma patients without and with an expert label for sepsis during follow-up, corresponding to our polytrauma case and controls, we further considered surgical admissions with sepsis already present on inclusion. Notably, among the non-polytrauma subgroups the incident sepsis subgroup was characterized by the highest proportion of men, severity of illness judged by SOFA and macrocirculatory abnormalities, and mortality (**Table 3**), consistent with sepsis development. The highest disease severity in this subgroup may explain the absence of differences in HVEM and ICOS compared to the sepsis on-inclusion subgroup (**Figure 7**). Yet, the absence of differences in both these two subgroups compared to the sepsis-free ICU controls is notable.

### 4.5 Limitations and strengths

We cannot fully exclude that the retrospective adjudication on the presence of sepsis by clinical experts may have been affected by hindsight bias, i.e., by the apprehended benefit for the patient of the therapeutic measures taken and of the clinical outcome achieved (66). For the timely identification of sepsis onset, however, this strategy is more appropriate than relying solely on clinical criteria for consensus definitions of sepsis. The latter was intended for different purposes, namely the inclusion of patients into trials to treat hyperinflammation (Sepsis-1) and retrospective epidemiological studies (Sepsis-3).

The major limitation of this study was the small number of almost exclusively male polytrauma patients in our cohort from only a single ICU. The limited statistical power precludes us from rejecting the absence of differences in study baseline blood levels in soluble immune checkpoints for candidates other than HVEM. However, delivery of care in our ICU over a reasonable enrollment period followed a consistent standard reducing the variance in iatrogenic risk factors for pneumonia and sepsis. Highly similar clinical characteristics support the comparability of the polytrauma post-injury pneumonia cases and infection-free controls.

A strength was the availability of close-meshed blood plasma samples with expert annotation on the presence of SIRS and sepsis according to Sepsis-1/2 between ICU-days two and six. This facilitated our trend analysis. Although we cannot extrapolate from our results to events beyond the follow-up period, we had previously shown that half of the overall sepsis cases in our ICU occur by day six (8) and in our polytrauma patients in particular by day seven (67). We also consider our conservative assessment of statistical significance by applying the correction for multiple testing and the absolute rather than relative quantitation of blood plasma concentrations as strengths.

## 5 Conclusions

The first major finding from this study was that the transition from *SIRS* to *sepsis*, but not to *severe sepsis* or *septic shock*, in critically ill patients by ICU-day six was associated with polytrauma as the reason for ICU admission and the lung as the site of infection. The second major finding consisted of continuously higher blood levels of HVEM in clinically highly similar polytrauma patients, who remained infection-free, compared to those who developed post-injury pneumonia during follow-up. At study inclusion, this difference was absent in surgical admissions developing sepsis during follow-up. HVEM may protect mucosal barrier function and, thereby, decrease the risk for secondary lung infection after polytrauma by increasing myeloid cell antimicrobial potential as a receptor of myeloid cell expressed LIGHT and/or by restricting lymphocyte-driven tissue damage as a ligand of T cell expressed BTLA.

Our data and their interpretation highlight the dual role of HVEM in appropriately balancing the immune response to violent tissue trauma and in modifying the associated risk for secondary pneumonia in respective ICU patients. It remains to be investigated whether reduced levels of HVEM after polytrauma mechanistically explain impaired lung immunity to infection or indirectly reflect an increased risk for pneumonia and whether a lower cut-off level for the prediction of sepsis in these patients can be determined.

## Data availability statement

Patient-level data on the clinical characteristics are not publicly available due to patient privacy but are available from the corresponding author upon reasonable request. The magnetic bead array results are available from heiDATA (https://heidata.uni-heidelberg.de/dataset.xhtml?persistentId=doi:10.11588/data/4Z4CY3) including (a) study baseline blood levels of HVEM and ICOS in polytrauma and non-polytrauma patients with and without sepsis, and (b) time trends of immune checkpoint blood levels in polytrauma patients with pneumonia and without infection.

## Ethics statement

The studies involving humans were approved by Medical Ethics Commission II of the Medical Faculty Mannheim, Heidelberg University, Germany (2016-643N-MA). The studies were conducted in accordance with the local legislation and institutional requirements. Written informed consent for participation in this study was provided by the participants’ legal guardians/next of kin.

## Author contributions

NS: Conceptualization, Data curation, Formal analysis, Investigation, Writing – original draft, Writing – review & editing. HL: Conceptualization, Funding acquisition, Investigation, Project administration, Supervision, Visualization, Writing – original draft, Writing – review & editing. BH: Data curation, Formal analysis, Writing – review & editing. RS: Formal analysis, Methodology, Visualization, Writing – review & editing. SV: Formal analysis, Writing – review & editing. JS: Data curation, Formal analysis, Methodology, Writing – review & editing. TF: Data curation, Formal analysis, Methodology, Writing – review & editing. FC: Data curation, Resources, Writing – review & editing. JS: Data curation, Resources, Writing – review & editing. BH: Formal analysis, Writing – review & editing. TS: Data curation, Resources, Writing – review & editing. MT: Funding acquisition, Project administration, Resources, Writing – review & editing. VS: Formal analysis, Supervision, Funding acquisition, Writing – original draft. AC: Conceptualization, Formal analysis, Investigation, Methodology, Project administration, Supervision, Writing – review & editing.
