# Supplementary material for: Pneumonia in the first week after polytrauma is associated with reduced blood levels of soluble herpes virus entry mediator
Source: Front Immunol. 2023 Dec 22;14:1259423. doi: 10.3389/fimmu.2023.1259423 (PMC10770833; doi:10.3389/fimmu.2023.1259423)
Supplement: Supplementary file 1 [file DataSheet_1.pdf]

Supplementary Material

Table of Contents

Item	Page
Supplementary Text 1	1
Supplementary Text 2	2
Supplementary Table 1	3
Supplementary Table 2	5
Supplementary Table 3	6
Supplementary Table 4	7
Supplementary Table 5	8
Supplementary Table 6	12
Supplementary Table 7	13
Supplementary Table 8	15
Supplementary Figure 1	22
Supplementary Figure 2	23
Supplementary Figure 3	24
Supplementary Figure 4	25
Supplementary Figure 5	26

Supplementary Text 1

Microbiology test results

Polytrauma post-injury pneumonia cases (N=7)

Microbiology testing in bronchoalveolar lavage samples identified Gram-negative bacteria in patients 9, 19, 44 and 63, and both Gram-negative and -positive bacteria in patients 27 and 66. Gram-positive bacteria were detected in blood cultures from patient 15.

Non-polytrauma with sepsis[1] on inclusion (N=19)

Microbiology test results were positive in only ten patients from this subgroup (53%). Blood cultures were positive for Gram-negative bacteria in one patient and Gram-positive bacteria in another. The latter also had Gram-positive bacteria in the sphenoid sinus and an intraabdominal swab. The swab also contained a fungus. Among the blood culture-negative patients, an intraabdominal swab, an abdominal surgical wound swab and ascites from one patient each were positive for Gram-negative bacteria. In four additional patients, fungal infections were detected in bronchoalveolar lavage. Lavage from two of these also contained Gram-negative and, from another patient, Gram-positive bacteria. Tracheal secretion of one additional patient tested positive for Gram-positive bacteria.

Non-polytrauma incident sepsis[1] cases (N=13)

Microbiology test results were positive in ten patients in this subgroup (77%). Both Gram-negative and -positive bacteria were detected in blood cultures from one and Gram-positive only from two patients, one of whom also had Gram-positive bacteria in tracheal secretion. Among the patients with negative blood cultures, one tested positive for Gram-positive, one for Gram-negative and one for both types of bacteria in tracheal secretions, and one for Gram-positive and one for Gram-negative bacteria in bronchoalveolar lavage. Gram-negative bacteria were detected in liquor from one further patient and Gram-positive in an abdominal swab from another.

[1] The presence of sepsis was adjudicated by clinical experts according to Sepsis-1/2.

Supplementary Text 2

Clinical characteristics of unselected non-polytrauma patients

The clinical characteristics of the unselected non-polytrauma are tabulated in Table 3 of the main text (mean plus standard deviation for selected values) and Supplementary Table 8 (median plus interquartile range for a more comprehensive set of values). They are summarized in the following. With 63.4 ± 16.7 years, the non-polytrauma patients were on average 16.1 years older than the polytrauma patients ($p = 0.004$), and there was a 2.3-fold relative excess of men in the incident sepsis[2] compared to the sepsis-free subgroup. In contrast to the polytrauma patients, they showed several further subgroup differences. For incident sepsis, relative hospital mortality was 2.9- and 3.7-times higher, respectively, than for patients included with sepsis and remaining sepsis-free. One patient with incident sepsis deceased after the fifth sampling time point, i.e., during follow-up. The overall mean hospital LOS for the non-polytrauma patients was 33 ± 28 days without subgroup differences. SOFA was highest in the incident sepsis subgroup. There were no neurosurgical patients in the sepsis on-inclusion subgroup. Diabetes (25%) and cardiovascular disease (50%) were the most common among the pre-existing chronic conditions, which overall showed no subgroup differences. Catecholamine therapy was more frequent in the incident sepsis cases (92%) than in patients remaining sepsis-free (54%) and with sepsis on-inclusion (58%). The latter had a negative median fluid balance compared to the other two subgroups ($p = 0.025$ for the comparison against incident sepsis). In incident sepsis cases, average systolic blood and mean arterial pressure, respectively, were lower than in patients with sepsis on inclusion ($p = 0.018$) and remaining sepsis-free ($p = 0.047$). Mean oxygen partial pressure (pO_2) and hemoglobin were higher and lower, respectively, in the sepsis-free compared to the sepsis on-inclusion subgroup ($p = 0.013$ for pO_2, $p = 0.049$ for hemoglobin). As signs of infection in sepsis on-inclusion cases, mean values for C-reactive protein (CRP) and proportions of neutrophils were elevated compared to the sepsis-free ($p = 0.001$) and the incident sepsis ($p = 0.008$) subgroup, respectively. Proportions of lymphocytes with sepsis on inclusion were lower than in both the incident sepsis ($p = 0.032$) and the sepsis-free ($p = 0.042$) subgroups.

[2] The presence of sepsis was adjudicated by clinical experts according to Sepsis-1/2.

Supplementary Table 1

List of MILLIPLEX map kits.

Soluble Immune Checkpoints
**Human Immuno-Oncology Checkpoint Protein Panel 1** (Cat. No. HCKPMAG-11K)[1]:<br>BTLA, CD27, CD28, CD40, CD80/B7-1, CD86/B7-2, CTLA-4/CD152, GITR, GITRL, HVEM, ICOS, LAG-3, PD-1, PD-L1, TIM-3, TLR-2
**Markers of Inflammation and Organ Dysfunction**
**Human Cytokine/Chemokine Panel I** (Cat. No. HCYTOMAG-60K):<br>Eotaxin/CCL11, G-CSF, IFN-alpha2, IL-12p40, IL-15, IL-1α, IL-1RA, IL-3, IP-10/CXCL10, PDGF-AA, PDGF-AB/BB, RANTES/CCL5, VEGF-A
**Human Cytokine/Chemokine Panel II** (Cat. No. HCYP2MAG-62K)<br>6Ckine/CCL21/Exodus-2, Eotaxin-3/CCL26
**Human Cytokine/Chemokine Panel III** (Cat. No. HCYP3MAG-63K)<br>MIG/CXCL9
**Human Cytokine/Chemokine Panel IV** (Cat. No. HCYP4MAG-64K):<br>HMGB1, IFNβ, IL-14/α-Taxilin
**Human High Sensitivity T Cell** (Cat. No. HSTCMAG-28SK):<br>Fractalkine/CX3CL1, GM-CSF, IFN-gamma, IL-10, IL-12p70, IL-13, IL-1β, IL-2, IL-21, IL-4, IL-6, IL-8/CXCL8, MIP-1α/CCL3, MIP-1β/CCL4, TNFα
**Human CD8+ T Cell** (Cat. No. HCYP4MAG-64K):<br>Granzyme A, Granzyme B, Perforin
**Human Soluble Cytokine Receptor** (Cat. No. HSCRMAG-32K)<br>sRAGE
**Human Complement Panel 1** (Cat. No. HCMP1MAG-19K):<br>C5a
**Human Sepsis Panel 1** (Cat. No. HSP1MAG-63K):<br>sFAS/TNFRSF6, sFasL, MIF, PAI-1 (total)
**Human Sepsis Panel 2** (Cat. No. HSP2MAG-63K):<br>HSP70
**Human Myokine** (Cat. No. HMYOMAG-56K)<br>Erythropoietin (EPO)

[1] A newer version of the Human Immuno-Oncology Checkpoint Protein Panel 1 (Cat. No. HCKP1-11K) additionally includes PD-L2 and was not yet available at the start of this analysis.

Supplementary Table 1 (continued)

List of MILLIPLEX map kits.

Markers of Inflammation and Organ Dysfunction
**Human CVD Panel 2** (Cat. No. HCVD2MAG-67K):<br>D-dimer, sICAM-1, MPO, sVCAM-1
**Human CVD Panel 3 (Acute Phase)** (Cat. No. HCVD3MAG-67K):<br>alpha-1-acid glycoprotein (AGP), Adipsin/Factor D, sL-Selectin, von Willebrand Factor (vWF)[2]
**Human CVD Panel 4** (Cat. No. HCVD4MAG-67K)<br>Tissue Factor
**Human CVD Panel 5** (Cat. No. HCVD5MAG-67K)[3]:<br>LDLR, SYND4
**Human Liver Injury** (Cat. No. HLINJMAG-75K):<br>ARG1, GSTα
**Human Angiogenesis Panel 2** (Cat. No. HANG2MAG-12K)<br>sIL-6Rα
**Human Kidney Injury Panel 4** (Cat. No. HKI4MAG-99K):<br>Renin
**Human Kidney Injury Panel 6** (Cat. No. HKI6MAG-99K):<br>Cystatin C

[2] The current version of Human CVD Panel 3 (Acute Phase) does not longer include vWF.
[3] Discontinued.

Supplementary Table 2

Details for hierarchical linear models of time trends in immune checkpoint levels in blood of polytrauma patients.

Immune checkpoint	Complete follow-up	Prior to sepsis onset
TIM-3	Random slope	Spatial covariance structure
CD27	Spatial covariance structure	Spatial covariance structure
CD40	Spatial covariance structure	Spatial covariance structure
HVEM	Spatial covariance structure	Random slope
GITR	Spatial covariance structure	Random slope
BTLA	Spatial covariance structure	Random slope
CTLA-4	Spatial covariance structure	Random intercept
PD-1	Spatial covariance structure	Random intercept
GITRL	Spatial covariance structure	Random intercept
CD80	Spatial covariance structure	Spatial covariance structure
CD28	Spatial covariance structure	Random slope
PD-L1	Random intercept	Random intercept
CD86	Random intercept	Random intercept
TLR2	Random intercept	Random intercept
ICOS	Spatial covariance structure	Random intercept
LAG3	Random intercept	Random intercept

Supplementary Table 3

Details on clinical scores in the polytrauma patients.

Patient group	Patient ID	CCI	ISS	RASS	TISS	SAPS II	SOFA
Infection-free controls	10	2	57	-5	22	20	15
	21	1	27	-5	22	26	10
	46	1	48	-5	23	38	13
	52	0	25	-3	23	41	4
	55	1	18	-5	22	21	9
	67	0	41	-5	27	17	11
	70	2	34	-5	18	21	10
Post-injury pneumonia cases	9	1	41	-5	18	35	16
	15	0	22	-1	10	29	5
	19	0	41	-5	22	32	13
	27	0	34	-5	22	23	9
	44	1	34	-4	15	33	7
	63	0	41	-5	22	21	12
	66	0	34	-5	22	22	10

The significance levels for patient group differences are indicated together with the mean values and standard deviations in Table 1 of the main text, and the p-values are given together with median values and interquartile ranges in Supplementary Table 5.

Supplementary Table 4

Details on blood transfusion therapies in the polytrauma patients between ICU admission (day 1) and study inclusion (day 2).

Patient group	Patient ID	Red blood cell concentrates (ml)	Fresh frozen plasma (ml)	Platelet concentrate transfusion (ml)	Fibrinogen replacement (g)
Infection-free controls	10	1200	2400	750	0
	21	0	0	0	0
	46	3900	12000	2700	17
	52	0	0	0	0
	55	0	0	0	0
	67	1500	0	0	0
	70	0	0	0	0
Post-injury pneumonia cases	9	0	0	0	0
	15	0	0	0	0
	19	0	2700	500	0
	27	0	1320	0	0
	44	0	0	0	0
	63	900	0	0	0
	66	0	0	0	0
P-palue[1]		0.32	0.90	0.62	0.71

[1] Mann-Whitney-Wilcoxon test (U test)

Supplementary Table 5

Clinical study baseline characteristics, ICU length of stay and mortality of polytrauma patients.

	**All polytrauma patients (N=14)**		**Infection-free controls (N=7)**		**Post-injury pneumonia cases (N=7)**		
	N	Median (IQR) N (%)	N	Median (IQR) N (%)	N	Median (IQR) N (%)	p-value
Group				7 (50.0%)		7 (50.0%)	
Age [yrs]	14	51 (33-55)	7	49 (33-55)	7	53 (26-56)	0.9267
Male		13 (92.9%)		7 (100%)		6 (85.7%)	1.0000~
LOS [days]	14	39.69 (22.8-65.7)	7	29.18 (22.8-86.8)	7	47.21 (20.9-65.7)	0.6160
Hospital mortality		0 (0%)		0 (0%)		0 (0%)	
**Admitting Department**							
Neurosurgery		2 (14.3%)		1 (14.3%)		1 (14.3%)	1.0000~
Orthopaedics and trauma centre		7 (50.0%)		3 (42.9%)		4 (57.1%)	1.0000~
Otorhinolaryngolo gy		1 (7.14%)		1 (14.3%)			
Surgery		6 (42.9%)		4 (57.1%)		2 (28.6%)	0.5921~
**Chronic Conditions**							
Diabetes		0 (0%)		0 (0%)		0 (0%)	
Cardiovascular diseases		3 (21.4%)		2 (28.6%)		1 (14.3%)	1.0000~
Respiratory diseases		0 (0%)		0 (0%)		0 (0%)	
Alcoholism		3 (21.4%)		2 (28.6%)		1 (14.3%)	1.0000~
Charlson Comorbidity Index	14	0.5 (0-1)	7	1 (0-2)	7	0 (0-1)	0.0756
**BGA and electrolytes**							
PO2 [mmHg]	14	84.9 (77.5-103)	7	87.5 (84.1-103)	7	77.5 (71.5-110)	0.5337

	All polytrauma patients (N=14)		Infection-free controls (N=7)		Post-injury pneumonia cases (N=7)		
	N	Median (IQR) N (%)	N	Median (IQR) N (%)	N	Median (IQR) N (%)	p-value
PCO2 [mmHg]	14	44.95 (43.1-47)	7	46.8 (42.6-55.6)	7	44.3 (43.1-45.2)	0.2223
ScvO2 [%]	12	76.65 (74.25-79.6)	7	76.7 (75-79.2)	5	75.7 (70.8-80)	0.4135
pH	14	7.411 (7.394-7.426)	7	7.425 (7.367-7.448)	7	7.403 (7.394-7.420)	0.9398
HCO3stand [mmol/L]	14	28.4 (27.4-31)	7	29.5 (27.4-33.4)	7	28.4 (26.2-28.5)	0.2000
BEstand [mmol/L]	14	4.1 (3.3-5.6)	7	5.1 (3.3-9.4)	7	3.7 (1.8-4.2)	0.2192
Na+ [mmol/L]	14	141 (137-142)	7	141 (140-143)	7	141 (133-142)	0.4655
K+ [mmol/L]	14	4 (3.8-4.1)	7	3.8 (3.5-4.1)	7	4 (4-4.3)	0.2377
Cl- [mmol/L]	14	107.5 (102-109)	7	106 (102-109)	7	109 (100-110)	0.7353
Ca++ [mmol/L]	14	1.11 (1.05-1.13)	7	1.13 (1.1-1.19)	7	1.09 (1.01-1.13)	0.3210
Lactate [mmol/L]	14	0.8 (0.7-1.5)	7	0.8 (0.8-2.7)	7	0.7 (0.5-1.5)	0.2934
SIDa [mmol/L]	14	38.6 (36.65-41.85)	7	39.15 (37.35-42.65)	7	36.95 (35.35-39.15)	0.2753
**Clinical chemistry**							
Creatinine [mg/dL]	14	1.08 (0.79-1.71)	7	1.09 (0.79-1.71)	7	0.88 (0.58-2.58)	0.7526
Urea [mg/dL]	14	34.05 (26.2-44.6)	7	34.7 (33.3-43.6)	7	31.7 (25.8-55.9)	0.9317
Glucose [mg/dL]	14	118.5 (110-135)	7	124 (110-139)	7	111 (107-129)	0.4449
Bilirubin [mg/dL]	14	0.83 (0.63-1.24)	7	0.79 (0.66-1.95)	7	0.97 (0.46-1.24)	0.8965
AST [U/L]	13	81 (46-274)	6	78 (62-274)	7	117 (31-306)	0.7816
ALT [U/L]	14	62.5 (33-216)	7	135 (39-591)	7	44 (26-106)	0.3867

	All polytrauma patients (N=14)		Infection-free controls (N=7)		Post-injury pneumonia cases (N=7)		
	N	Median (IQR) N (%)	N	Median (IQR) N (%)	N	Median (IQR) N (%)	p-value
Lipase [U/L]	14	85.5 (64-207)	7	91 (70-207)	7	76 (51-210)	0.5912
CRP [mg/L]	14	175.5 (130-252)	7	164 (102-223)	7	189 (130-253)	0.4107
PCT [µg/L]	9	0.82 (0.27-4)	4	11.77 (0.79-41.625)	5	0.27 (0.26-0.98)	0.2495
**Hematology**							
Hb [g/dL]	14	8.85 (7.9-10.2)	7	9 (8.4-10.2)	7	8.7 (7.6-10.8)	0.6729
WBC [10E9/L]	14	10.98 (8.72-12.45)	7	9.03 (5.59-12.45)	7	12.14 (10.72-13.95)	0.1509
INR	14	1.07 (1.02-1.12)	7	1.07 (1.07-1.12)	7	1.07 (0.99-1.19)	0.5120
Thrombocytes [10E9/L]	14	156.5 (91-213)	7	156 (90-213)	7	157 (106-213)	0.7821
Basophils [%]	14	0.15 (0.1-0.2)	7	0.1 (0.1-0.2)	7	0.2 (0.1-0.3)	0.2959
Eosinophils [%]	14	0.3 (0.1-1)	7	0.3 (0.1-0.7)	7	0.7 (0-1.6)	0.2868
Neutrophils [%]	14	83.9 (74.1-85.1)	7	84.1 (72.8-85)	7	83.7 (74.1-87.5)	0.9532
Lymphocytes [%]	14	9.3 (6.8-15.8)	7	9.3 (9.2-13.9)	7	9 (5.8-16.8)	0.8352
Monocytes [%]	14	6.3 (5.8-9)	7	6.2 (6-9)	7	6.4 (5.7-9.4)	0.5556
**Vital signs / Clinical characteristics**							
Temperature [°C]	14	37.65 (37.1-37.8)	7	37.3 (37-37.7)	7	37.7 (37.1-37.8)	0.2526
Respiratory rate [1/min]	14	21 (20-24)	7	20 (17-22)	7	22 (20-24)	0.1366
Heart rate [1/min]	14	93.5 (84-99)	7	92 (84-94)	7	99 (77-111)	0.3823
Systolic blood pressure [mmHg]	14	121 (108-131)	7	117 (104-131)	7	125 (108-131)	0.6800

	All polytrauma patients (N=14)		Infection-free controls (N=7)		Post-injury pneumonia cases (N=7)		
	N	Median (IQR)<br>N (%)	N	Median (IQR)<br>N (%)	N	Median (IQR)<br>N (%)	p-value
Diastolic blood pressure [mmHg]	14	59<br>(55-73)	7	58<br>(52-60)	7	63<br>(57-73)	0.3022
Mean arterial pressure [mmHg]	14	80.5<br>(76-90)	7	77<br>(70-90)	7	84<br>(78-90)	0.3716
Shock index	14	0.78<br>(0.73-0.88)	7	0.76<br>(0.72-0.88)	7	0.82<br>(0.74-0.92)	0.8716
SpO2 [%]	14	98.5<br>(94-100)	7	99<br>(97-100)	7	97<br>(94-100)	0.6508
Mechanical ventilation (y)		14 (100%)		7 (100%)		7 (100%)	
FiO2	14	0.3<br>(0.3-0.35)	7	0.3<br>(0.3-0.3)	7	0.35<br>(0.3-0.4)	0.0353
Horovitz Index [mmHg]	14	280.33<br>(226.9-343.3)	7	291.67<br>(275.0-343.3)	7	226.86<br>(177.3-366.7)	0.2812
Fluid balance [mL]	14	15.59<br>(-18.4-82.1)	7	17.96<br>(-25.2-104.9)	7	13.21<br>(-15.5-82.1)	0.8308
Pressor therapy (norepinephrine or dobutamine, y)		10 (71.4%)		5 (71.4%)		5 (71.4%)	1.0000~
**Clinical Scores**							
ISS	14	34<br>(27.0-41.0)	7	34<br>(25.5-46.3)	7	34<br>(34.0-41.0)	0.9429
RASS	14	-5<br>(-5--5)°	7	-5<br>(-5--5)°	7	-5<br>(-5--4)°	0.5160
TISS	14	22<br>(18-22)	7	22<br>(22-23)	7	22<br>(15-22)	0.1008
SAPS II	14	24.5<br>(21-33)	7	21<br>(20-38)	7	29<br>(22-33)	0.7153
SOFA	14	10<br>(9-13)	7	10<br>(9-13)	7	10<br>(7-13)	1.0000

All data apply to study inclusion except for ICC (ICU admission).
p-value: t-test (method Satterthwaite) for continuous parameters
#: Mann-Whitney-Wilcoxon test (U test)
Chi² test for categorical parameters
~: Fisher's exact test
° mode [interquartile range (IQR)]
Admitting department: more than one department per patient possible

Supplementary Table 6

Supporting literature for immune checkpoint receptor-ligand pairs.

Receptor-ligand pairs	References
HVEM-BTLA	Sedy JR, Gavrieli M, Potter KG, Hurchla MA, Lindsley RC, Hildner K, Scheu S, Pfeffer K, Ware CF, Murphy TL et al: B and T lymphocyte attenuator regulates T cell activation through interaction with herpesvirus entry mediator. *Nature immunology* 2005, 6(1):90-98.
GITR-GITRL	Kwon B, Yu KY, Ni J, Yu GL, Jang IK, Kim YJ, Xing L, Liu D, Wang SX, Kwon BS: Identification of a novel activation-inducible protein of the tumor necrosis factor receptor superfamily and its ligand. *J Biol Chem* 1999, 274(10):6056-6061.<br>Gurney AL, Marsters SA, Huang RM, Pitti RM, Mark DT, Baldwin DT, Gray AM, Dowd AD, Brush AD, Heldens AD et al: Identification of a new member of the tumor necrosis factor family and its receptor, a human ortholog of mouse GITR. *Curr Biol* 1999, 9(4):215-218.
CTLA4-CD80,<br>CTLA4-CD86,<br>CD80-CD28,<br>CD28-CD86	Linsley PS, Greene JL, Brady W, Bajorath J, Ledbetter JA, Peach R: Human B7-1 (CD80) and B7-2 (CD86) bind with similar avidities but distinct kinetics to CD28 and CTLA-4 receptors. *Immunity* 1994, 1(9):793-801.
PD-1-PD-L1	Freeman GJ, Long AJ, Iwai Y, Bourque K, Chernova T, Nishimura H, Fitz LJ, Malenkovich N, Okazaki T, Byrne MC et al: Engagement of the PD-1 immunoinhibitory receptor by a novel B7 family member leads to negative regulation of lymphocyte activation. *J Exp Med* 2000, 192(7):1027-1034.

Supplementary Table 7

Estimates for the intercept (study baseline value) and slope (hourly change) from hierarchical linear models of time trends of soluble immune checkpoints in blood plasma of polytrauma patients before sepsis onset and statistical evaluations after Bonferroni adjustment.

**Immune Checkpoint**	**Polytrauma post-injury pneumonia cases**				**Polytrauma infection-free controls**				**Subgroup differences**	
	Intercept (pg/mL)	Slope [pg/mL/h] (95% CI)	Relative hourly change [% of intercept]	P-value slope	Intercept (pg/mL)	Slope [pg/mL/h] (95% CI)	Relative hourly change [% of intercept]	P-value slope	P-value intercept difference	P-value slope difference
TIM-3	4542	29.64 (3.75 – 55.53)	0.65	0.451	5154	32.91 (10.93 – 54.89)	0.64	0.109	1.000	1.000
CD27	2677	-6.96 (-24.24 – 10.32)	-0.26	1.000	3206	26.76 (12.85 – 40.66)	0.83	**0.020**	1.000	**0.035**
CD40	702	-0.4 (-3.96 – 3.16)	-0.06	1.000	814	1.2 (-1.78 – 4.19)	0.15	1.000	1.000	1.000
HVEM	2472	1.23 (-10.17 – 12.63)	0.05	1.000	3728	4.8 (-4.71 – 14.31)	0.13	1.000	**0.030**	1.000
GITR	166	0.21 (-0.91 – 1.34)	0.13	1.000	102	-0.84 (-1.83 – 0.15)	-0.83	1.000	1.000	1.000
BTLA	838	0.74 (-5.02 – 6.50)	0.09	1.000	860	-5.69 (-10.83 – -0.55)	-0.66	0.525	1.000	1.000
CTLA-4	151	0.11 (-0.49 – 0.71)	0.07	1.000	123	-0.7 (-1.19 – -0.22)	-0.57	0.086	1.000	0.628
PD-1	997	0.20 (-3.42 – 3.82)	0.02	1.000	890	-3.54 (-6.42 – -0.67)	-0.4	0.272	1.000	1.000
GITRL	597	-0.04 (-1.71 – 1.63)	-0.01	1.000	396	-1.42 (-2.74 – -0.09)	-0.36	0.589	1.000	1.000
CD80	175	-0.03 (-0.77 – 0.72)	-0.02	1.000	151	-0.68 (-1.30 – -0.06)	-0.45	0.544	1.000	1.000

Immune Checkpoint	Polytrauma post-injury pneumonia cases				Polytrauma infection-free controls				Subgroup differences	
	Intercept (pg/mL)	Slope [pg/mL/h] (95% CI)	Relative hourly change [% of intercept]	P-value slope	Intercept (pg/mL)	Slope [pg/mL/h] (95% CI)	Relative hourly change [% of intercept]	P-value slope	P-value intercept difference	P-value slope difference
CD28	6616	9.39 (-14.78 – 33.57)	0.14	1.000	5526	-19.97 (-38.51 – -1.43)	-0.36	0.590	1.000	0.703
CD86	1107	-0.65 (-6.21 – 4.90)	-0.06	1.000	1188	-5.38 (-9.60 – -1.16)	-0.45	0.221	1.000	1.000
PD-L1	108	-0.03 (-0.67 – 0.60)	-0.03	1.000	150	-0.72 (-1.21 – -0.23)	-0.48	0.076	1.000	1.000
TLR-2	2111	1.20 (-5.72 – 8.12)	0.06	1.000	2297	-4.28 (-9.37 – 0.81)	-0.19	1.000	1.000	1.000
ICOS	734	-0.29 (-7.45 – 6.88)	-0.04	1.000	1826	-8.34 (-13.85 – -2.83)	-0.46	0.064	**0.044**	1.000
LAG-3	24807	-43.41 (-152.54 – 65.73)	-0.17	1.000	31419	24.4 (-68.63 – 117.44)	0.08	1.000	1.000	1.000

Supplementary Table 8

Clinical study baseline characteristics, ICU length of stay and mortality of non-polytrauma patients.

	Total (N=56)		SIRS (N=24)		Sepsis[a] on inclusion (N=19)		Incident sepsis[a] (N=13)		Sepsis[a] on inclusion vs. SIRS	Incident sepsis[a] vs. SIRS	Incident sepsis[a] vs. sepsis[a] on inclusion
	N	Median (IQR) N (%)	N	Median (IQR) N (%)	N	Median (IQR) N (%)	N	Median (IQR) N (%)	p-value	p-value	p-value
Group				24 (42.9%)		19 (33.9%)		13 (23.2%)			
Age [yrs]	56	65 (54.5-77)	24	63 (59.5-74.5)	19	64 (44-79)	13	71 (55-77)	0.6141	0.7731	0.4829
Male		30 (53.6%)		9 (37.5%)		10 (52.6%)		11 (84.6%)	0.3211	0.0060	0.1279~
LOS [days]	56	24.25 (16.0-39.1)	24	24.50 (16.1-38.3)	19	28.09 (17.7-46.0)	13	23.09 (10.3-32.3)	0.4046	0.6636	0.3003
Hospital mortality		16 (28.6%)		4 (16.7%)		4 (21.1%)		8 (61.5%)	1.0000~	0.0097~	0.0300~
**Admitting Department**											
Neurosurgery		14 (25.0%)		10 (41.7%)				4 (30.8%)	0.0013	0.5141	0.0097
Orthopaedics and trauma centre		6 (10.7%)		2 (8.33%)		3 (15.8%)		1 (7.69%)	0.6404~	1.0000~	0.6291~
Other		9 (16.1%)		3 (12.5%)		5 (26.3%)		1 (7.69%)			
Otorhino–laryngology		8 (14.3%)		3 (12.5%)		4 (21.1%)		1 (7.69%)			

	Total (N=56)		SIRS (N=24)		Sepsis[a] on inclusion (N=19)		Incident sepsis[a] (N=13)		Sepsis[a] on inclusion vs. SIRS	Incident sepsis[a] vs. SIRS	Incident sepsis[a] vs. sepsis[a] on inclusion
	N	Median (IQR) N (%)	N	Median (IQR) N (%)	N	Median (IQR) N (%)	N	Median (IQR) N (%)	p-value	p-value	p-value
Surgery		23 (41.1%)		9 (37.5%)		8 (42.1%)		6 (46.2%)	0.7590	0.6088	0.8206
**Chronic Conditions**											
Diabetes		14 (25.0%)		8 (33.3%)		5 (26.3%)		1 (7.69%)	0.6188	0.1193~	0.3606~
Cardiovascular diseases		28 (50.0%)		15 (62.5%)		9 (47.4%)		4 (30.8%)	0.3211	0.0653	0.3477
Respiratory diseases		3 (5.36%)		0 (0%)		2 (10.5%)		1 (7.69%)	0.1894~	0.3514~	1.0000~
Alcoholism		2 (3.57%)		1 (4.17%)		1 (5.26%)		0 (0%)	1.0000~	1.0000~	1.0000~
Charlson Comorbidity Index	56	2 (1-3)	24	2 (1-3)	19	2 (1-4)	13	1 (1-3)	0.9042	0.8073	0.8809
**BGA and electrolytes**											
PO2 [mmHg]	56	84.4 (76.65-101.5)	24	94.3 (77.6-105)	19	78.1 (72.1-91.6)	13	87.9 (79.4-112)	0.0129	0.9458	0.0512
PCO2 [mmHg]	56	41.8 (38.5-47.6)	24	41.85 (37.4-48.1)	19	41.3 (39.4-43.2)	13	44.4 (36.8-49.6)	0.9032	0.7001	0.6567
ScvO2 [%]	37	72 (67.9-76.3)	15	72.3 (67.9-75)	12	70.05 (63.8-75.7)	10	72.75 (69.9-78.7)	0.4556	0.6627	0.3279

	Total (N=56)		SIRS (N=24)		Sepsis[a] on inclusion (N=19)		Incident sepsis[a] (N=13)		Sepsis[a] on inclusion vs. SIRS	Incident sepsis[a] vs. SIRS	Incident sepsis[a] vs. sepsis[a] on inclusion
	N	Median (IQR) N (%)	N	Median (IQR) N (%)	N	Median (IQR) N (%)	N	Median (IQR) N (%)	p-value	p-value	p-value
pH	56	7.420 (7.367-7.460)	24	7.433 (7.395-7.470)	19	7.421 (7.360-7.455)	13	7.390 (7.370-7.433)	0.5913	0.1848	0.4023
HCO3stand [mmol/L]	56	27.05 (24.35-29.5)	24	27.2 (25.95-29)	19	26.9 (24.3-29.7)	13	27.1 (21.9-30.4)	0.6558	0.3874	0.6398
BEstand [mmol/L]	56	3.05 (0.1-5.55)	24	3.15 (2.15-5)	19	3.1 (-0.4-6)	13	2.2 (-1.9-5.1)	0.6466	0.2682	0.5084
Na+ [mmol/L]	56	141 (137.5-143.5)	24	141 (138.5-143)	19	142 (136-148)	13	141 (138-143)	0.7364	0.4820	0.3636
K+ [mmol/L]	56	4.1 (3.9-4.3)	24	4.1 (3.9-4.3)	19	4.1 (3.9-4.3)	13	4.2 (4.1-4.5)	0.8930	0.6157	0.5757
Cl- [mmol/L]	56	107 (106-111)	24	107 (105.5-111.5)	19	108 (103-112)	13	109 (106-111)	0.9545	0.5517	0.6525
Ca++ [mmol/L]	56	1.11 (1.07-1.16)	24	1.12 (1.09-1.18)	19	1.11 (1.06-1.15)	13	1.1 (1.07-1.15)	0.2752	0.2756	0.9190
Lactate [mmol/L]	56	1 (0.8-1.55)	24	1 (0.8-1.6)	19	1 (0.7-1.6)	13	0.9 (0.7-1.4)	0.5369	0.5697	0.4654
SIDa [mmol/L]	56	38.85 (34.4-40)	24	37.5 (35.3-40)	19	39.35 (34.15-40.95)	13	38.05 (34.25-39.45)	0.4106	0.7016	0.2790

	**Total (N=56)**		**SIRS (N=24)**		**Sepsis[a] on inclusion (N=19)**		**Incident sepsis[a] (N=13)**		**Sepsis[a] on inclusion vs. SIRS**	**Incident sepsis[a] vs. SIRS**	**Incident sepsis[a] vs. sepsis[a] on inclusion**
	N	Median (IQR) N (%)	N	Median (IQR) N (%)	N	Median (IQR) N (%)	N	Median (IQR) N (%)	p-value	p-value	p-value
**Clinical chemistry**											
Creatinine [mg/dL]	54	0.935 (0.66-1.48)	24	0.98 (0.645-1.305)	17	0.89 (0.75-2.56)	13	0.88 (0.65-1.68)	0.1546	0.4524	0.4651
Urea [mg/dL]	54	45.55 (33.6-62.9)	24	43.2 (28.55-59.35)	17	46.2 (41.8-86)	13	47.1 (34.4-55.4)	0.0719	0.3463	0.4262
Glucose [mg/dL]	56	136.5 (109-170)	24	143.5 (113.5-177)	19	127 (98-155)	13	134 (114-162)	0.1191	0.4761	0.4376
Bilirubin [mg/dL]	51	0.49 (0.32-0.85)	23	0.47 (0.27-0.88)	16	0.55 (0.33-1)	12	0.505 (0.365-0.81)	0.9018	0.3869	0.4257
AST [U/L]	51	40 (27-70)	23	38 (24-78)	16	44 (27.5-56)	12	51 (27.5-72.5)	0.2401	0.4446	0.2221
ALT [U/L]	51	33 (19-58)	23	33 (16-41)	16	39.5 (20.5-67.5)	12	23.5 (20-62.5)	0.4088	0.8699	0.3962
Lipase [U/L]	51	91 (50-200)	23	137 (64-241)	16	81.5 (60-175.5)	12	58 (47-129)	0.2729	0.7943	0.4553
CRP [mg/L]	54	141.5 (85.9-181)	24	124 (47.75-155)	17	178 (142-322)	13	141 (85.9-181)	0.0014	0.1678	0.0541

	Total (N=56)		SIRS (N=24)		Sepsis[a] on inclusion (N=19)		Incident sepsis[a] (N=13)		Sepsis[a] on inclusion vs. SIRS	Incident sepsis[a] vs. SIRS	Incident sepsis[a] vs. sepsis[a] on inclusion
	N	Median (IQR) N (%)	N	Median (IQR) N (%)	N	Median (IQR) N (%)	N	Median (IQR) N (%)	p-value	p-value	p-value
PCT [µg/L]	38	0.485 (0.22-1.32)	13	0.39 (0.22-0.6)	15	1.13 (0.3-6.18)	10	0.235 (0.12-1.08)	0.0981	0.4627	0.1263
**Hematology**											
Hb [g/dL]	56	8.8 (8.2-10.05)	24	8.65 (8.1-9.4)	19	9.2 (8.5-10.8)	13	8.8 (8.2-9.2)	0.0494	0.9792	0.0675
WBC [10E9/L]	54	12.30 (9.21-14.90)	24	12.69 (9.81-14.72)	17	12.73 (10.12-16.84)	13	9.45 (6.48-12.32)	0.9445	0.2833	0.2991
INR	54	1.045 (1-1.1)	24	1.04 (1.005-1.07)	17	1.06 (1-1.12)	13	1.01 (0.96-1.1)	0.2266	0.8733	0.3339
Thrombocytes [10E9/L]	54	174.5 (116-252)	24	148.5 (103.5-221)	17	211 (183-292)	13	140 (105-242)	0.1358	0.8893	0.1122
Basophils [%]	38	0.2 (0.1-0.3)	20	0.2 (0.1-0.2)	10	0.2 (0.1-0.3)	8	0.25 (0.15-0.3)	0.8643	0.3293	0.4185
Eosinophils [%]	38	0.15 (0-0.8)	20	0.1 (0-0.45)	10	0.2 (0-0.6)	8	0.65 (0.2-1.3)	0.4862	0.9168	0.4107
Neutrophils [%]	38	82.45 (78-87.7)	20	83 (76.85-87.45)	10	87.4 (79.8-90.7)	8	80.45 (76.95-81.95)	0.0778	0.2386	0.0075
Lymphocytes [%]	38	6.85 (4.7-10.9)	20	7.05 (5.6-12.15)	10	4.45 (4.2-6.3)	8	9.15 (7.25-11.15)	0.0416	0.4404	0.0315

	Total (N=56)		SIRS (N=24)		Sepsis[a] on inclusion (N=19)		Incident sepsis[a] (N=13)		Sepsis[a] on inclusion vs. SIRS	Incident sepsis[a] vs. SIRS	Incident sepsis[a] vs. sepsis[a] on inclusion
	N	Median (IQR) N (%)	N	Median (IQR) N (%)	N	Median (IQR) N (%)	N	Median (IQR) N (%)	p-value	p-value	p-value
Monocytes [%]	38	7.4 (5.8-11.2)	20	7.6 (5.2-11.1)	10	7 (4.9-7.8)	8	9.45 (7.2-11.85)	0.4744	0.3380	0.1591
**Vital signs / Clinical characteristics**											
Temperature [°C]	55	37.1 (36.8-37.5)	23	37.1 (36.8-37.5)	19	37 (36.6-37.5)	13	37.1 (36.8-37.4)	0.9370	0.6515	0.7542
Respiratory rate [1/min]	56	18 (15-22)	24	17 (14.5-22)	19	19 (16-22)	13	18 (16-22)	0.5208	0.2689	0.5316
Heart rate [1/min]	56	89.5 (73.5-100)	24	89.5 (77-98.5)	19	89 (68-101)	13	91 (71-106)	0.6232	0.8197	0.5671
Systolic blood pressure [mmHg]	56	124.5 (113.5-139)	24	127.5 (108.5-144)	19	129 (120-141)	13	115 (106-128)	0.4686	0.1111	0.0179
Diastolic blood pressure [mmHg]	56	60 (52.5-68)	24	63.5 (51-68)	19	61 (53-78)	13	56 (53-60)	0.9302	0.2246	0.2605
Mean arterial pressure [mmHg]	56	80 (73-93.5)	24	82 (75.5-95)	19	80 (74-98)	13	73 (68-83)	0.9086	0.0469	0.0988
Shock index	56	0.67 (0.60-0.83)	24	0.68 (0.60-0.86)	19	0.64 (0.50-0.77)	13	0.78 (0.62-0.90)	0.2892	0.3737	0.0921

	Total (N=56)		SIRS (N=24)		Sepsis[a] on inclusion (N=19)		Incident sepsis[a] (N=13)		Sepsis[a] on inclusion vs. SIRS	Incident sepsis[a] vs. SIRS	Incident sepsis[a] vs. sepsis[a] on inclusion
	N	Median (IQR) N (%)	N	Median (IQR) N (%)	N	Median (IQR) N (%)	N	Median (IQR) N (%)	p-value	p-value	p-value
SpO2 [%]	56	97.5 (95-100)	24	99 (95.5-100)	19	96 (93-99)	13	97 (96-99)	0.0606	0.3981	0.3111
Mechanical ventilation (y)		47 (83.9%)		20 (83.3%)		15 (78.9%)		12 (92.3%)	1.0000~	0.6378~	0.6247~
FiO2	52	0.325 (0.3-0.4)	23	0.3 (0.3-0.4)	16	0.35 (0.3-0.475)	13	0.3 (0.3-0.4)	0.6091	0.6370	0.2946
Horovitz Index [mmHg]	56	254.24 (202.0-338.0)	24	261.25 (232.5-354.4)	19	201.25 (191.4-309.0)	13	263.71 (219.8-304.7)	0.1079	0.4402	0.4922
Fluid balance [mL]	56	16.02 (-71.4-80.4)	24	17.26 (-71.4-83.7)	19	-8.00 (-171.7-68.0)	13	50.17 (0.0-83.0)	0.1036	0.1400	0.0250
Pressor therapy (norepinephrine or dobutamine, y)		36 (64.3%)		13 (54.2%)		11 (57.9%)		12 (92.3%)	0.8069	0.0272~	0.0497~
**Clinical Scores**											
RASS	55	0 (-4-0)°	24	0 (-3-0)°	18	0 (-3-0)°	13	-4 (-4--1)°	0.6913	0.4087	0.5998
TISS	56	15 (10-18.5)	24	14 (10-18.5)	19	13 (10-18)	13	18 (14-22)	0.6479	0.1465	0.0694

	Total (N=56)		SIRS (N=24)		Sepsis[a] on inclusion (N=19)		Incident sepsis[a] (N=13)		Sepsis[a] on inclusion vs. SIRS	Incident sepsis[a] vs. SIRS	Incident sepsis[a] vs. sepsis[a] on inclusion
	N	Median (IQR) N (%)	N	Median (IQR) N (%)	N	Median (IQR) N (%)	N	Median (IQR) N (%)	p-value	p-value	p-value
SAPS II	56	35.5 (29-44)	24	35 (28.5-40)	19	37 (28-44)	13	36 (33-48)	0.6059	0.3573	0.6263
SOFA	56	7 (5-10)	24	7 (4-9.5)	19	6 (5-7)	13	11 (6-12)	0.3527	0.0538	0.0122

All data apply to study inclusion.
[a]Sepsis-1/2
p-value: t-test (method Satterthwaite) for continuous parameters, Chi² test for categorical parameters
#: Mann-Whitney-Wilcoxon test (U test), ~: Fisher's exact test, °: mode (IQR)

Supplementary Figure 1

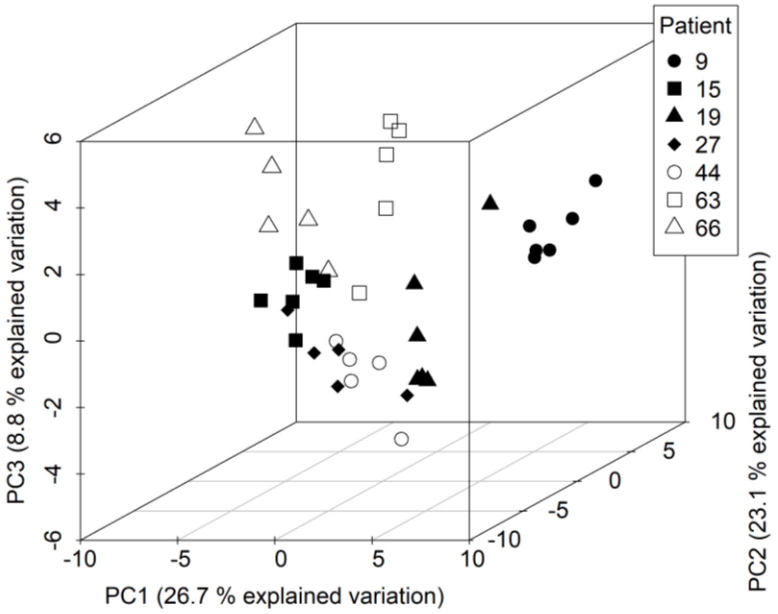

Principal component analysis of serial blood plasma samples from seven polytraumatized patients developing pneumonia during follow-up based on the concentrations of immune checkpoints and markers of inflammation and organ dysfunction (see Figure 3 of the main manuscript). Percentages represent the variance captured by the first three principal components (PC1, PC2, and PC3).

Supplementary Figure 2

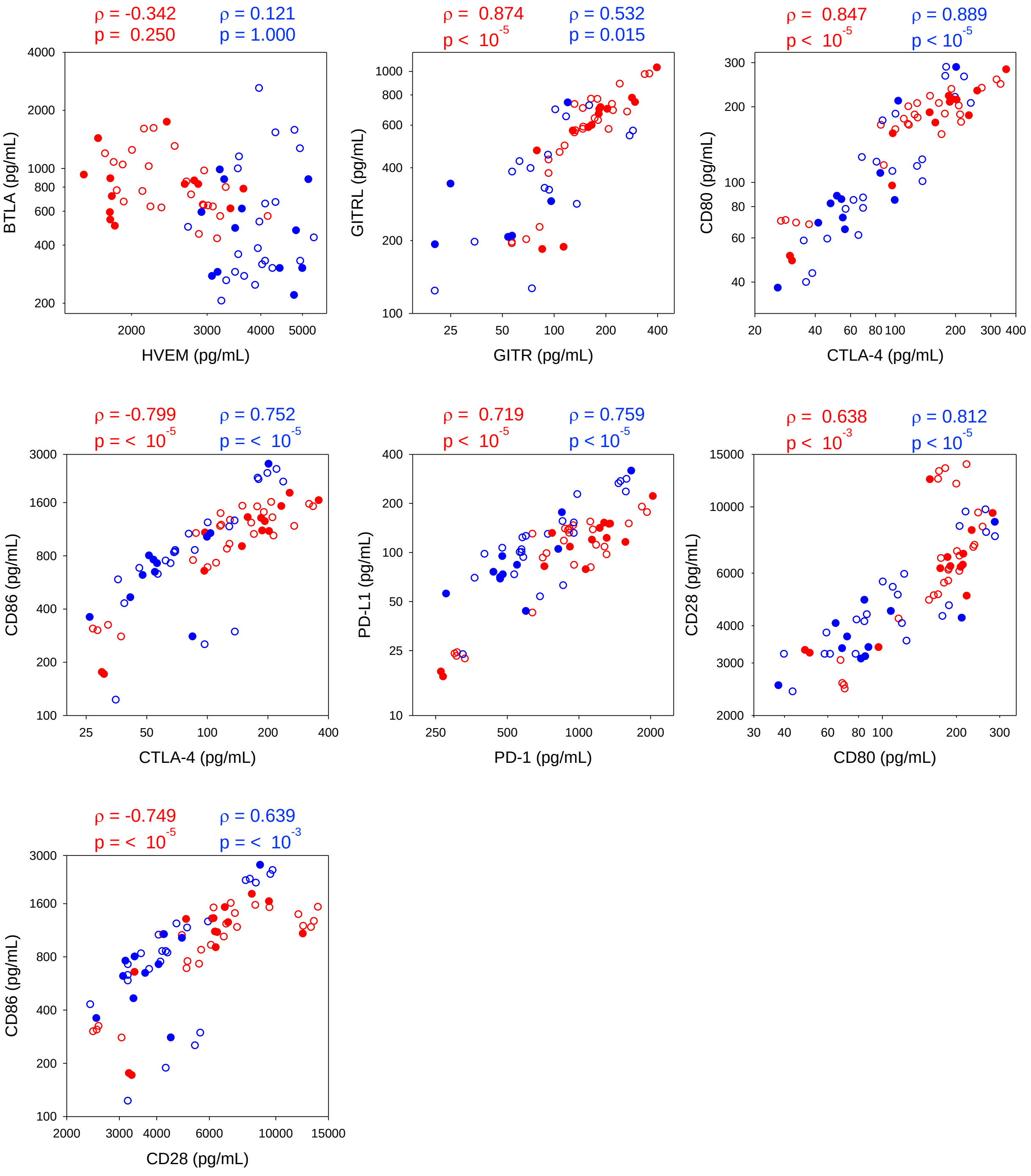

Spearman's rank-order correlation for blood plasma concentrations of immune checkpoint receptors and their putative ligands in the polytrauma patients. Data for the seven post-injury pneumonia cases are shown on a log scale in red and for the seven age-matched infection-free controls in blue. Determinations on time points in cases without and with sepsis labels are shown as red open and closed circles, respectively. Determinations on reference time points in controls are represented accordingly. P-values were Bonferroni-adjusted.

Supplementary Figure 3

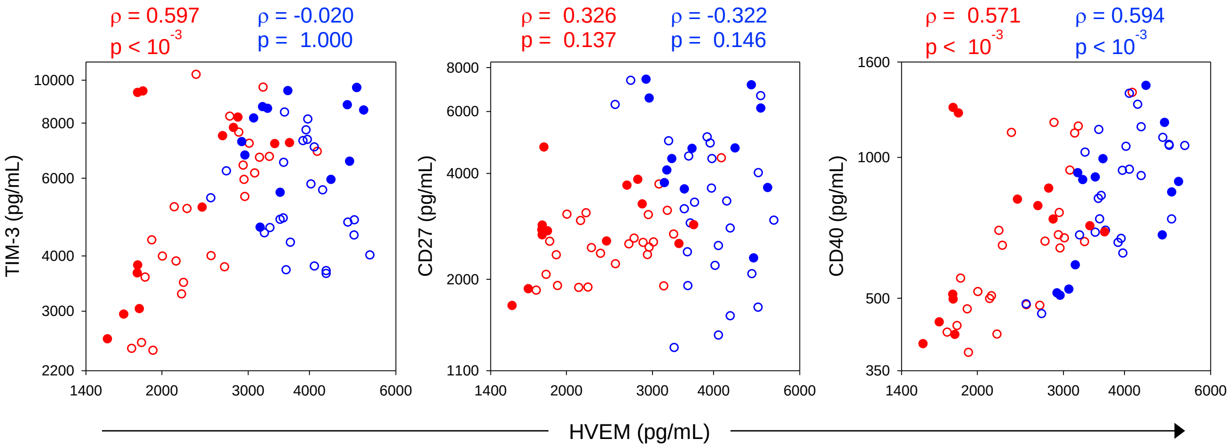

Spearman's rank-order correlation for blood plasma concentrations of HVEM with the other subcluster A immune checkpoints in the polytrauma patients. Data for the seven post-injury pneumonia cases are shown on a log scale in red and for the seven age-matched sepsis-free controls in blue. Determinations on time points in cases (red) without and with sepsis labels are shown as red open and closed circles, respectively. Determinations on reference time points in controls (blue) are represented accordingly. P-values were Bonferroni-adjusted.

Supplementary Figure 4

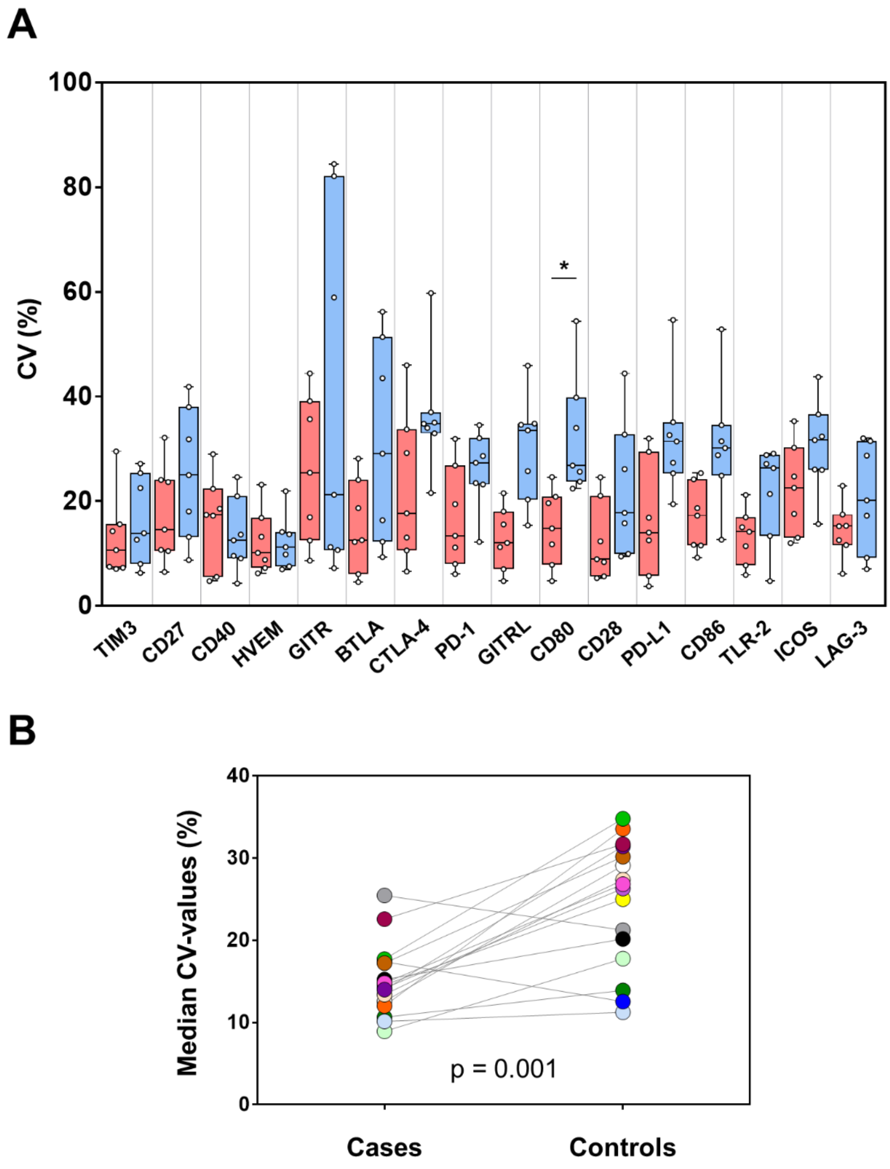

Variation in blood plasma immune checkpoint concentrations in the seven polytrauma post-injury pneumonia cases and the seven age-matched infection-free polytrauma controls. **A** Coefficient of variation (CV)-values from all immune checkpoint determinations during follow-up in cases (red boxes) and controls (blue boxes) are plotted side-by-side for each checkpoint as box plots with whiskers from minimum to maximum and overlaid with the CV-values for individual patients as dispersion plots. The subgroup difference was statistically significant only for CD80. *p = 0.032 from Mann-Whitney U test followed by Bonferroni adjustment. **B** The medians of the CV-values for all cases and controls and for each immune checkpoint, plotted individually in panel A, are summarized as dot plot. Dots for same checkpoints in the two patient subgroups are connected by lines. The nevertheless independent data was compared with the Mann-Whitney U test with the resultant p-value indicated.

Supplementary Figure 5

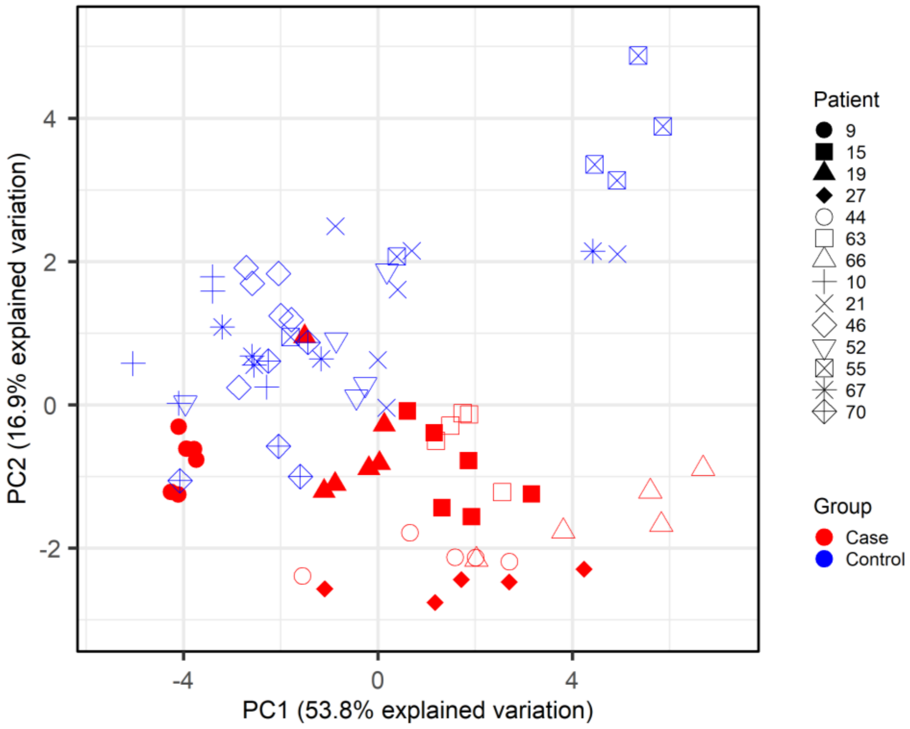

Principal component analysis plot for the seven polytrauma post-injury pneumonia cases and matched controls based on the blood plasma concentrations of 16 immune checkpoints. Percentages represent the variance captured by the first two principal components (PC1 and PC2).
